# AAV Exploits Subcellular Stress Associated with Inflammation, Endoplasmic Reticulum Expansion, and Misfolded Proteins in Models of Cystic Fibrosis

**DOI:** 10.1371/journal.ppat.1002053

**Published:** 2011-05-19

**Authors:** Jarrod S. Johnson, Martina Gentzsch, Liqun Zhang, Carla M. P. Ribeiro, Boris Kantor, Tal Kafri, Raymond J. Pickles, R. Jude Samulski

**Affiliations:** 1 Department of Pharmacology, University of North Carolina, Chapel Hill, North Carolina, United States of America; 2 Gene Therapy Center, University of North Carolina, Chapel Hill, North Carolina, United States of America; 3 Cystic Fibrosis Research Center, University of North Carolina, Chapel Hill, North Carolina, United States of America; 4 Department of Cell and Developmental Biology, University of North Carolina, Chapel Hill, North Carolina, United States of America; 5 Department of Medicine, University of North Carolina, Chapel Hill, North Carolina, United States of America; 6 Department of Microbiology and Immunology, University of North Carolina, Chapel Hill, North Carolina, United States of America; King's College London School of Medicine, United Kingdom

## Abstract

Barriers to infection act at multiple levels to prevent viruses, bacteria, and parasites from commandeering host cells for their own purposes. An intriguing hypothesis is that if a cell experiences stress, such as that elicited by inflammation, endoplasmic reticulum (ER) expansion, or misfolded proteins, then subcellular barriers will be less effective at preventing viral infection. Here we have used models of cystic fibrosis (CF) to test whether subcellular stress increases susceptibility to adeno-associated virus (AAV) infection. In human airway epithelium cultured at an air/liquid interface, physiological conditions of subcellular stress and ER expansion were mimicked using supernatant from mucopurulent material derived from CF lungs. Using this inflammatory stimulus to recapitulate stress found in diseased airways, we demonstrated that AAV infection was significantly enhanced. Since over 90% of CF cases are associated with a misfolded variant of Cystic Fibrosis Transmembrane Conductance Regulator (ΔF508-CFTR), we then explored whether the presence of misfolded proteins could independently increase susceptibility to AAV infection. In these models, AAV was an order of magnitude more efficient at transducing cells expressing ΔF508-CFTR than in cells expressing wild-type CFTR. Rescue of misfolded ΔF508-CFTR under low temperature conditions restored viral transduction efficiency to that demonstrated in controls, suggesting effects related to protein misfolding were responsible for increasing susceptibility to infection. By testing other CFTR mutants, G551D, D572N, and 1410X, we have shown this phenomenon is common to other misfolded proteins and not related to loss of CFTR activity. The presence of misfolded proteins did not affect cell surface attachment of virus or influence expression levels from promoter transgene cassettes in plasmid transfection studies, indicating exploitation occurs at the level of virion trafficking or processing. Thus, we surmised that factors enlisted to process misfolded proteins such as ΔF508-CFTR in the secretory pathway also act to restrict viral infection. In line with this hypothesis, we found that AAV trafficked to the microtubule organizing center and localized near Golgi/ER transport proteins. Moreover, AAV infection efficiency could be modulated with siRNA-mediated knockdown of proteins involved in processing ΔF508-CFTR or sorting retrograde cargo from the Golgi and ER (calnexin, KDEL-R, β-COP, and PSMB3). In summary, our data support a model where AAV exploits a compromised secretory system and, importantly, underscore the gravity with which a stressed subcellular environment, under internal or external insults, can impact infection efficiency.

## Author Summary

Misfolded proteins have been associated with a variety of disorders such as cystic fibrosis, diabetes insipidus, alpha-antitrypsin deficiency, Parkinson's disease, and cancer. In this study, by using cellular models of events in cystic fibrosis lung disease we have revealed an effect of misfolded proteins on increasing susceptibility to infection with a parvovirus. Infection efficiency was an order of magnitude higher in cells expressing misfolded Cystic Fibrosis Transmembrane Conductance Regulator (CFTR) mutant proteins than in cells expressing the correctly folded protein. During infection, virus capsids accumulated near cellular factors that normally process misfolded proteins and are involved in retrograde trafficking from the Golgi to endoplasmic reticulum. Furthermore, we have demonstrated that infection efficiency can be attenuated by restoring correct protein folding or augmented by siRNA-mediated knockdown of secretory pathway components. Taken together our results indicate that converging cellular systems operate to clear misfolded proteins and virus capsids from an infected cell. We raise the possibility that parvoviruses and perhaps other viruses exploit congested cellular secretory pathways during entry, and that viral infection could be a contributing factor in the progression of diseases associated with misfolded proteins.

## Introduction

Evolutionary biology speaks of ‘survival of the fittest’. A dark twist to this principle is that often the survival of an organism depends on its exploitation of another. This relationship is vividly apparent in virology, as many viruses have evolved as opportunistic pathogens and take advantage of hosts under stress. One attractive hypothesis is that this exploitation can occur at subcellular levels when stresses associated with inflammation, ER expansion, or misfolded proteins are present. Therefore, in this study we have explored whether pleiotropic subcellular stressors and effects related to misfolded protein processing will mitigate barriers to infection using models of cystic fibrosis (CF).

CF is the most common lethal progressive hereditary disorder in Caucasian populations [1] and manifests primarily in the lungs. Afflicted individuals are plagued by mucus accumulation, airway inflammation, and persistent infections. This monogenic disorder is typically linked to mutations in the Cystic Fibrosis Transmembrane Conductance Regulator (CFTR). CFTR is a cyclic adenosine monophosphate-activated chloride ion channel that primarily functions at the apical surface of airway epithelium. Loss of normal CFTR function impairs regulation of chloride transport across the airway surface, which leads to a reduction of airway surface liquid volume, airway mucus dehydration, decreased mucus transport, and increased risk of airway infection.

A single deletion of a phenylalanine residue at position 508 in CFTR (ΔF508-CFTR) results in a misfolded protein that is associated with approximately 90% of CF cases [2]. The ΔF508 mutation disrupts the proper folding of CFTR during transit in the cellular secretory pathway, thus incorrectly limiting its glycosylation and trafficking to the plasma membrane. When the secretory pathway identifies ΔF508-CFTR as misfolded it is rapidly degraded in the cell through ER-associated degradation [3], [4]. Historically, degradation of misfolded CFTR was thought to occur primarily via ubiquitin-proteasome degradation pathways [5], [6], but recent evidence suggests autophagy may also play a role [7]. While controversy exists over whether effects related to ΔF508-CFTR misfolding play a role in disease progression, it has been clearly documented that CF cells experience subcellular stress as indicated by increased sensitivity to Ca^2+^-mediated inflammation, ER-expansion, and activation of the unfolded protein response [8], [9]. Independent of an exogenous inflammatory stimulus, overexpression of ΔF508-CFTR *in vitro* causes ER stress, activates an unfolded protein response [10], and is a well-established model for studying subcellular effects of the misfolded protein [11].

Misfolded proteins and pathogens that rely on cellular machinery for their life-cycles use a common trafficking pathway that sorts them to the microtubule organizing center (MTOC) where they can be targeted for degradation by either the ubiquitin/proteasome system or the autophagosome/lysosome [12], [13], [14]. Also known as the centrosome, the MTOC is the point of origin from which microtubules emanate outward toward the cell periphery. This network of microtubules provides a scaffold for the organization of key vesicles and organelles such as endosomes, lysosomes, the Golgi apparatus, and the nucleus [15]. Retrograde trafficking along microtubules may be beneficial to viruses during entry, as it has the potential to place the infectious material in close proximity to the nucleus. However, the high concentration of proteasomes and lysosomes in this region [16], [17] may serve to expedite degradation and clearance of pathogen components from the cell; thus, restricting infection. During subcellular stress, the degradation and sorting capacity at the MTOC can be exceeded, which can result in proteasome dysfunction, accumulation of protein aggregates, formation of aggresomes, expansion of the secretory pathway, and activation of the unfolded protein response [18]. Based on these findings, we hypothesized that cells under stress due to misfolded proteins might be more susceptible to virus infection.

For these studies we tested if subcellular stress and expression of misfolded proteins could be exploited by adeno-associated virus (AAV) and promote increased infection efficiency. AAV is a non-enveloped parvovirus and classified as a *dependovirus*, as it will only enter a replicative state when specific conditions are obtained in the host cell, such as during coinfection with a helper virus like adenovirus or herpes virus [19]. It has previously been shown that the most highly characterized serotype, AAV2, is inefficient at delivering its genome to the nucleus, with the majority of virions remaining outside the nuclear membrane [20], [21], [22]. After cell surface binding, AAV2 infection progresses through clathrin- and dynamin-dependent internalization into an endosomal compartment [23], [24], [25]. Capsids have been detected in the early endosome, recycling endosome, late endosome, lysosome, and Golgi apparatus [26]. Although many details of AAV infection remain to be elucidated, it represents an attractive virus for testing our hypothesis since AAV2 undergoes retrograde trafficking on microtubules [27] and infection is negatively affected by the ubiquitin/proteasome system [28]. Indeed, it has been shown that proteasome inhibitors potentiate AAV-mediated gene delivery by up to several hundred fold [21], [22], [28], [29]. Therefore, it seems likely that AAV virions may be handled in a manner similar to misfolded proteins, and following pharmacological interference or saturation of these pathways, the cell would be less equipped to block virus trafficking and be rendered more susceptible to infection.

Here, we demonstrate that cells under subcellular stress are more susceptible to viral infection. Cells expressing ΔF508-CFTR were more susceptible to AAV transduction by an order of magnitude over cells expressing wild-type CFTR. This effect was not due to differences in AAV cell-surface attachment or changes in reporter gene expression, since no significant variation was observed in virus binding studies or in expression levels of plasmid reporters from transfection studies. Our results suggest that the exploitation of cellular entry by AAV is at the level of virus trafficking or capsid processing through a direct or indirect relationship with components of the secretory pathway. These findings connect stresses associated with misfolded proteins and the secretory system to diminishing subcellular barriers to infection.

## Results

### Human ciliated airway epithelial cell (HAE) cultures under subcellular stress are rendered more susceptible to AAV infection

**Figure 1:**
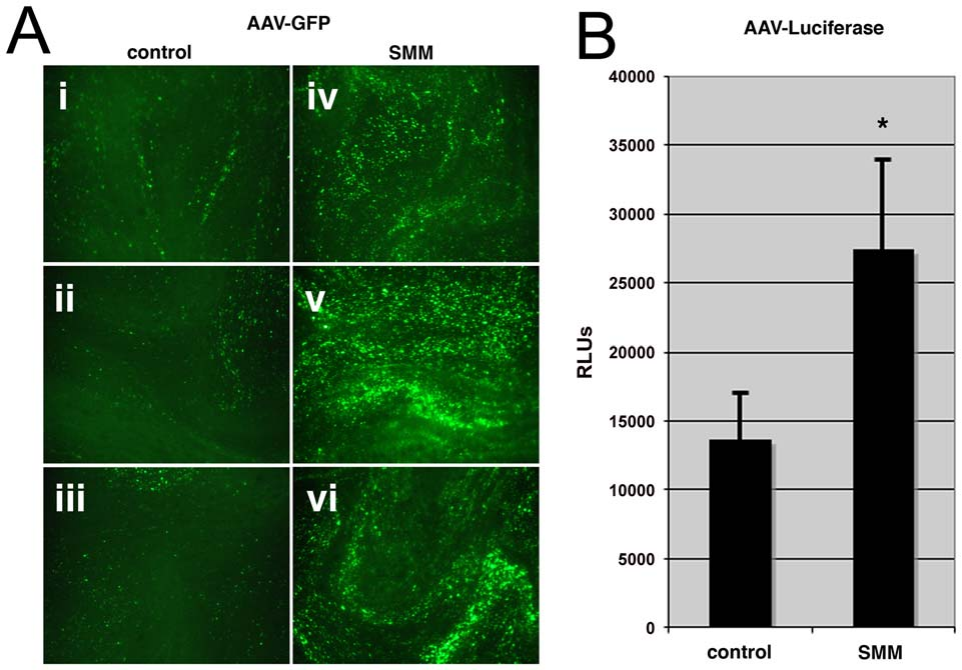
Human airway epithelium under stress is more susceptible to AAV infection. HAE cultures were exposed to SMM an inflammatory stimulant derived from CF airways by application to the apical surface to induce cellular stress and model diseased airway. 24 hr after application, SMM was removed and HAE infected with an AAV variant generated by molecular directed evolution. A. Representative en face images of three HAE cultures one week after inoculation with AAV packaging an eGFP transgene, showing GFP positive cells (green) in HAE cultures exposed to PBS (panels i, ii, and iii) or SMM (iv, v, and vi). B. Parallel experiments performed on HAE cultures with an AAV packaging a Luc transgene. Cells were harvested one week after inoculation and luciferase activity was assessed. Error bars represent standard deviations from three separate inoculations. Data shown are representative from three independent experiments (* p-value<0.05).

It has previously been demonstrated that the subcellular status of the CF airway epithelium displays an increase in sensitivity to Ca^2+^-mediated inflammation, expansion of the ER, and activation of the unfolded protein response [8], [9], [30]. These conditions can be reproduced in HAE cultures derived from excised lung tissue by apical administration of an inflammatory stimulus; supernatant from mucopurulent material (SMM) [8], [9], derived from CF airways. We used SMM to model the inflammatory state of HAE under stress and tested whether these conditions increase the susceptibility of HAE to AAV transduction. SMM or a PBS control was applied to the apical surface of HAE grown at an air-liquid interface. After 24 hrs of SMM treatment, HAE cultures were inoculated at the apical surface with an AAV variant (HAE-2), carrying transgene reporters for either enhanced green fluorescent protein (GFP) or firefly luciferase (Luc), that we have previously generated by molecular directed evolution [31]. As described by Li et al., transduction could be detected by one week post-inoculation [31], although we found significantly more GFP-positive cells in HAE treated with SMM prior to AAV inoculation (Fig. 1A, panels iv, v, and vi), compared to controls (panels i, ii, and iii). Quantification in parallel cultures using AAV expressing luciferase showed that although the difference in luciferase activity between cultures treated with PBS or SMM was not as great as that for GFP, transduction in SMM-treated samples was 2-fold higher than controls (Fig. 1B) and found to be statistically significant (p-value<0.05). For the remainder of these studies we have employed use of the prototypic serotype, AAV2, which has been more thoroughly characterized than the variant, HAE-2. Since it is difficult to detect transduction of AAV2 in airway epithelium cultures, for this experiment it was more appropriate to utilize the AAV variant that exhibits the capacity for greater transduction in these cells.

### Cells expressing ΔF508 CFTR are more permissive to viral infection

**Figure 2:**
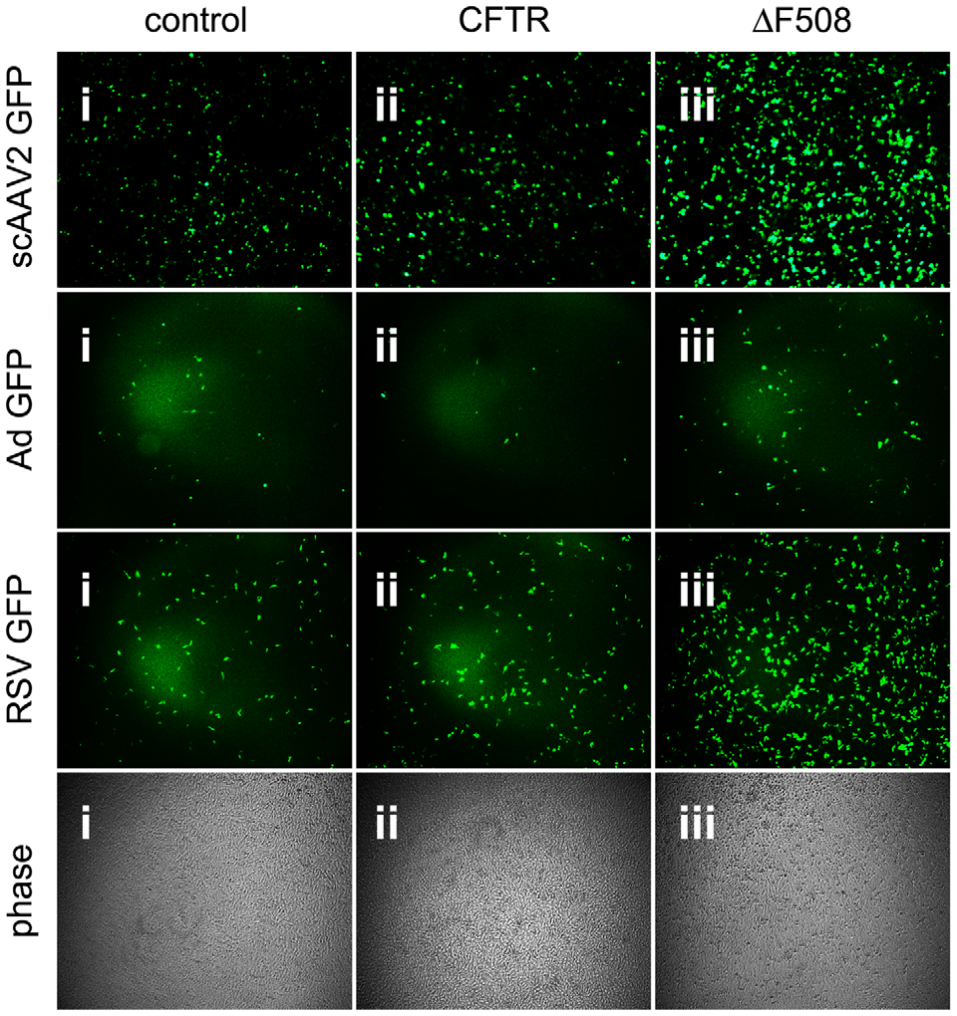
Cells expressing misfolded ΔF508-CFTR are more permissive to infection with human respiratory viruses. Images of GFP-positive cells after inoculation with recombinant self-complementary AAV (scAAV2, 100 vg/cell: eGFP transgene), adenovirus (Ad; 20 vg/cell), or respiratory syncytial virus (RSV; 200 vg/cell) into control BHK-21 cells (panel i), BHK-21 cells overexpressing CFTR (ii), or BHK-21 cells overexpressing ΔF508-CFTR (iii). Representative phase contrast images are shown to demonstrate equivalent levels of confluence.

**Figure 3:**
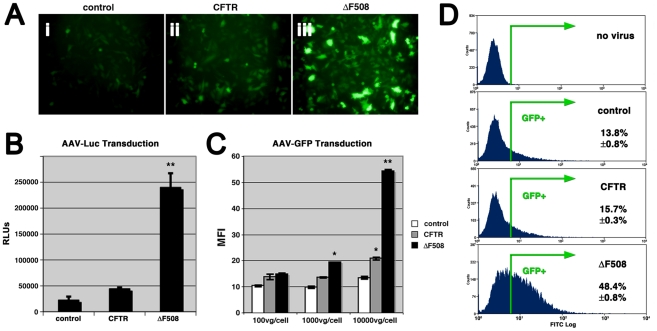
Cells expressing misfolded ΔF508-CFTR are more permissive to AAV infection. A. Images of GFP-positive cells after administration of recombinant AAV (AAV2, 1,000 vg/cell: eGFP transgene) to BHK-21 cells (panel i), BHK-21 cells overexpressing CFTR (ii), or BHK-21 cells overexpressing ΔF508-CFTR (iii). B. Luciferase activity of cells after transduction by AAV (AAV2, 1,000 vg/cell: Luc transgene) in control BHK-21 cells, or cells expressing CFTR, or expressing ΔF508-CFTR. C. Flow cytometry analysis of AAV transduction at increasing particle numbers showing mean fluorescence intensity (MFI) of GFP expression from positive cells after inoculation with AAV2-GFP at 100, 1000, or 10000 vg/cell. D. Plots show percent of GFP-positive cells after infection with AAV2-GFP (1,000 vg/cell) in control BHK-21 cells, CFTR cells, or ΔF508-CFTR cells. Samples that were statistically different from controls are marked (*p<0.05; **p<0.01).

Using an inflammatory stimulant to induce subcellular stress in HAE cultures has pluripotent consequences that may affect changes in addition to expansion of the secretory pathway and activation of the unfolded protein response. Since the majority of CF disease is associated with misfolded ΔF508-CFTR [2], we sought to explore whether expression of misfolded proteins could independently increase infection efficiency of AAV2 or other human respiratory viruses. To this end, stable lines of control wild-type BHK-21 cells, BHK-21 cells overexpressing CFTR, or BHK-21 cells overexpressing ΔF508-CFTR, were inoculated with self-complementary AAV2 (scAAV2), adenovirus (Ad), or respiratory syncytial virus (RSV) packaging GFP reporter genes (Fig. 2). Transduction was noticeably higher for all three viruses in cells expressing ΔF508-CFTR. We chose to continue our analysis of this exploitation of misfolded ΔF508-CFTR using AAV as a model opportunistic respiratory virus. Similar to what was observed for scAAV2, 24 hr after inoculation of single-stranded rAAV2, the expression of GFP (Fig. 3A) or Luc (Fig. 3B) reporters indicated that infection was significantly more efficient in ΔF508-CFTR-expressing cells than in control or CFTR-overexpressing BHK-21 cells. Similar data to that shown for BHK-21 cells was also obtained in other cell lines such as CHO and HEK-293, with higher AAV2 transduction efficiencies measured in ΔF508-CFTR-expressing cells as compared to controls (wild-type BHK-21 cells or CFTR-expressing cells, data not shown).

Luciferase assays suggested that AAV transduction efficiency was augmented 10-fold in cells expressing ΔF508-CFTR over controls, yet it is difficult to determine from this approach alone whether the enhanced efficiency stems from an increase in the number of cells transduced or an increase in expression intensity. To more clearly assess this enhancement, we analyzed cells inoculated with AAV-GFP by flow cytometry. GFP expression levels were similar at low virion numbers [100 vector genomes per cell (vg/cell)], according to the mean fluorescence intensity (MFI) values acquired from GFP-positive cells (Fig. 3C). Thus, transcription, translation, and transgene expression levels from one infectious event likely did not differ much between control, CFTR, and ΔF508-CFTR cells. At higher virion numbers, GFP expression levels per cell were significantly higher in ΔF508 cells (Fig. 3C), suggesting the threshold for multiple infectious events is lower in cells expressing the misfolded protein. Consistent with this finding, we determined that the number of GFP-positive cells was greater in ΔF508-CFTR cells than in controls (Fig. 3D); [AAV2-GFP (1000 vg/cell) transduced 48.4%±0.8% of ΔF508-CFTR cells, compared to 13.8%±0.8% and 15.7%±0.3%, of control and CFTR cells, respectively]. This improvement in the number of cells transduced by AAV was similar over a range of particle numbers, from 100 vg/cell to 10000 vg/cell and was observed for two different promoters: TR-CMV-GFP and TR-CBA-GFP (data not shown).

### Effect of low temperature on processing of ΔF508-CFTR and transduction by AAV

**Figure 4:**
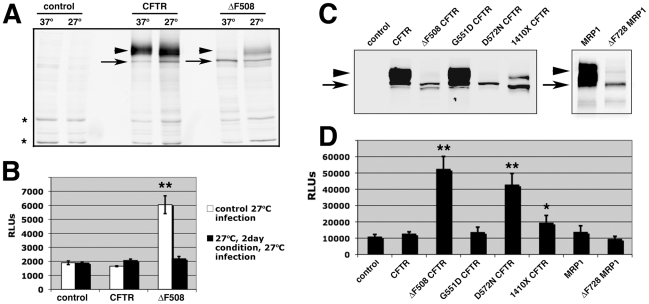
Effect of low temperature conditioning or expression of other misfolded proteins on AAV2 infection. A. Western blot showing immature form of CFTR and ΔF508 (black arrow) and mature glycosylated form (arrowhead). Following low temperature conditioning (2 d, 27°C), a significant amount of ΔF508-CFTR becomes more complexly glycosylated. Asterisks (*) represent non-specific banding and demonstrate equal loading. B. Luciferase assay of transduction after low temp conditioning, and a comparative control at 27°C. Cells were infected with AAV2 (10,000 vg/cell) as indicated and luciferase activity was measured 24 hr later. C. Comparative analysis of mutant CFTR processing with AAV2 transduction. Western blot of proteins expressed in BHK-21 cell lines depicting level of glycosylation of CFTR, and mutants ΔF508 (misfolded), G551D (properly folded), D572N (misfolded), 1410× (intermediate folding defect). Related ABC transporter proteins MRP1 and an analogous mutant ΔF728-MRP1 are also shown. D. Luciferase assay for transduction in these cell lines following administration of AAV2 (10,000 vg/cell). Samples that were statistically different from controls are indicated with p-values marked (*p<0.05; **p<0.01).

ΔF508-CFTR folding and trafficking defects can be rescued by culturing cells under low temperature (27°C) conditions [32], [33]. If the repercussions from processing misfolded proteins benefits viral infection, then reducing the misfolded protein burden by low temperature conditioning should mitigate the infectious advantage observed in ΔF508-CFTR cells. To test this notion, we assayed AAV infection under low temperature conditions in control BHK-21 cells and cells overexpressing CFTR or ΔF508-CFTR after 48 hrs of maintaining the cells at 27°C or 37°C. We first determined whether our protocol resulted in corrected trafficking of ΔF508-CFTR by performing western blot analysis. Figure 4A shows that after two days of low-temperature conditioning at 27°C, a considerable amount of ΔF508-CFTR protein is correctly glycosylated and runs at the predicted molecular mass (∼180 kD) indicating a rescued phenotype similar to wild-type CFTR. We noted that overall levels of infection efficiency at 27°C are below what is observed at 37°C (compare Fig. 4B with Fig. 3B). Nonetheless, we observed AAV2 transduction efficiency to be at least 3-fold higher in ΔF508-CFTR cells than in control cells and CFTR cells (Fig. 4B, white columns) when these cells were initially held at 37°C. In contrast, after preconditioning cells at 27°C, we observed no differences in AAV2 transduction levels between control, CFTR, and ΔF508-CFTR cells (Fig. 4B, black columns), suggesting that enhanced transduction efficiency in ΔF508-expressing cells was lost once the protein was allowed to fold correctly and traffic to the cell membrane.

### Comparative analysis of mutant CFTR processing with AAV2 transduction

In addition to performing ΔF508-CFTR rescue experiments, we explored whether increased infection efficiency in the presence of ΔF508-CFTR was a generalized phenomenon that could be reproduced with other misfolded proteins. To this end, we tested AAV transduction efficiency in BHK-21 cells lines expressing CFTR or ΔF508-CFTR compared to cell lines expressing other mutant CFTR proteins and related ABC transporters. The G551D (glycine to aspartic acid) CFTR mutant folds properly and traffics to the plasma membrane, but does not display an operative chloride ion channel [34]. When AAV2 was administered to cell lines expressing G551D-CFTR (Fig. 4C), no significant difference in transduction was observed compared to control or CFTR-expressing cells (Fig. 4D). We also tested two other CFTR mutants that, like ΔF508-CFTR, do not traffic to the membrane (Fig. 4C). D572N (aspartic acid to asparagine) CFTR is misfolded, rapidly cleared from the cell, and cannot be rescued even at low temperature, whereas the truncated 1410X-CFTR mutant shows a partial defect in trafficking [35]. Based on measured levels of transduction, AAV is able to exploit expression of D572N-CFTR to levels similar to that achieved in ΔF508-CFTR-expressing cells. However, the effect of 1410X-CFTR expression on AAV transduction was intermediate between that measured in ΔF508-CFTR and control cells. It is interesting to note that the level of transduction enhancement corresponded to the degree of misfolding for each of the CFTR mutants, as indicated by the ratio of immature (mass) to mature (mass) glycosylated forms of protein (Fig. 4C). Related ABC transporter proteins MRP1 and an analogous mutant to ΔF508-CFTR (ΔF728-MRP1) do not share the misfolding and degradation pattern of ΔF508-CFTR [36] and when expressed in BHK-21 cells do not render cells more susceptible to AAV2 transduction. Together, these data suggest that AAV is able to exploit cells expressing a variety of misfolded proteins. Moreover, this exploitation is not related to loss of wild-type CFTR activity at the plasma membrane, since G551D-CFTR-expressing cells did not show increased AAV transduction even in the absence of ion channel function.

### Cell-surface attachment of AAV to control, CFTR, and ΔF508-CFTR expressing cells is similar

Virions could potentially take advantage of cells under stress at a number of steps in the infectious pathway. To test whether expression of misfolded ΔF508 CFTR renders cells more susceptible to infection due to an increase in cell surface attachment of virus particles, we performed a binding assay with AAV2 on these cell lines (supplementary Fig. S1A). Equal numbers of control wild-type BHK-21 cells (purple diamonds), cells expressing CFTR (red squares), or ΔF508-CFTR (blue triangles) cooled to 4°C in order to inhibit endocytosis. While at this temperature, AAV2 virions were administered to the cell medium (10,000 vg/cell) for 5, 10, 20, or 60 min. Subsequently, unbound virus particles and medium were washed from the cells and the remaining cell-associated vector genomes were isolated by DNA purification. Vector genome amounts were quantified by qPCR and no significant differences were observed in binding rates, indicating that AAV2 likely exploits expression of ΔF508-CFTR at steps in the infectious pathway after cellular attachment.

### Analysis of expression levels after transfection of reporter plasmids

To determine whether ΔF508-CFTR expression influenced transcription, translation, or reporter protein stability, we exogenously introduced GFP or Luc reporter plasmids into wild-type BHK-21 cells or cells expressing CFTR or ΔF508-CFTR by transfection and quantified expression levels by flow cytometry and luciferase assay 24 hr later. It should be noted these were the same promoter-transgene cassettes that were used for virus production. Expression of GFP after transfection was within the same order of magnitude among control cells, cells expressing CFTR, or cells expressing ΔF508 (supplementary Fig. S1B). Results from luciferase assays were similar to those from flow cytometry, (supplementary Fig. S1C). Transfection of reporter plasmids into CFTR-expressing cells yielded higher GFP and Luc expression intensities, and transfection into ΔF508-expressing cells resulting in slightly lower reporter gene expression levels than those seen with CFTR. In the GFP study, but not for Luc, these differences were found to have a p-value of less than 0.01, yet in both cases the differences were less than two-fold. While not dramatically different, the observation that general expression levels after transfection were higher in CFTR-expressing cells than in ΔF508 cells might imply a further distinction in how significantly ΔF508 cells are more susceptible to viral transduction. From these experiments it is reasonable to conclude that AAV virions are able to exploit cells expressing misfolded ΔF508-CFTR at steps after receptor binding but prior to vector gene expression during infection.

### Localization of AAV capsids in control, CFTR, or ΔF508-CFTR cells

**Figure 5:**
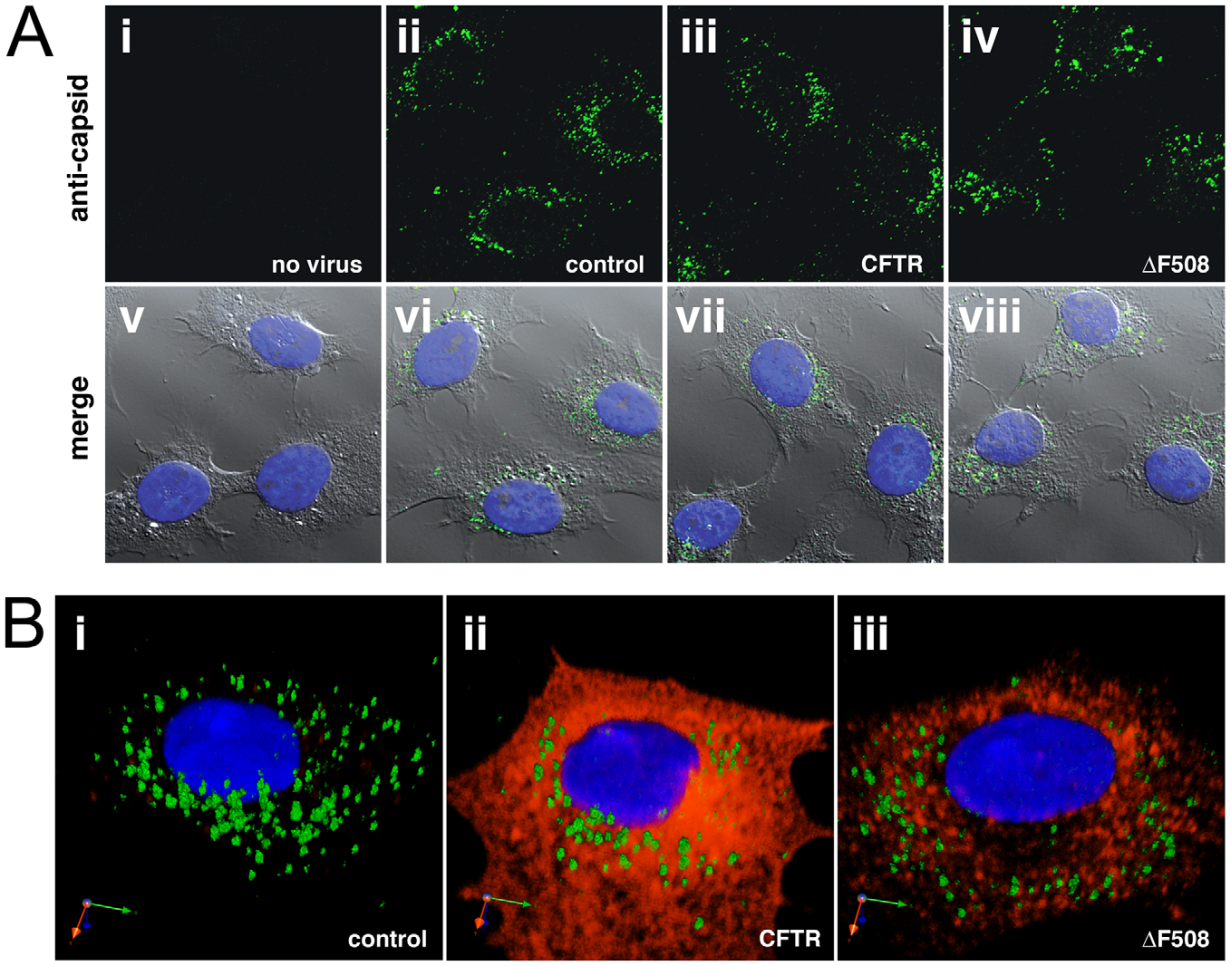
Perinuclear accumulation of AAV capsids in control, CFTR, or ΔF508-CFTR cells. A. Confocal immunofluorescence staining of capsids (green) 16 hr post-inoculation (50,000 vg/cell) do not demonstrate obvious differences in subcellular trafficking at this level of resolution. B. Single cell 3D composites were rendered using volume imaging software on z-stacked images taken after co-staining virus capsids with CFTR (panel ii, red) or ΔF508-CFTR (iii, red). Absence of yellow color suggests no direct interaction between AAV and wt or mutant CFTR, yet does not rule out that they could be degraded or processed through the same pathway. Nuclei (blue) were stained with DAPI.

To explore the possibility that ΔF508-CFTR has an observable effect on the trafficking profiles of AAV2 capsids, we followed AAV trafficking by confocal immunofluorescence microscopy during infection. In BHK-21 cells, at no time point after inoculation was there a clear distinction between localization of capsids (green) in control cells, CFTR cells, and ΔF508-expressing cells (16 hr time point shown, Fig. 5A). BHK-21 cells that were not infected with virus were processed identically to other samples and demonstrate no background antibody staining (Fig. 5A, panel i). Capsid staining displayed the characteristic perinuclear ring in all cell types, with various amounts of punctate or aggregated capsid signal surrounding the nucleus (blue). At this level of resolution there did not appear to be a significant difference in capsid trafficking among these cell types, but the possibility remains that capsids could be processed differently in ΔF508-CFTR cells, which could result in more efficient or expedited delivery of the transgene to the nucleus.

Mutant ΔF508-CFTR is usually recognized by quality control proteins in the secretory pathway as being misfolded and is extracted into the cytoplasm and subject to ER-associated degradation through the ubiquitin/proteasome system [37]. With this in mind, we analyzed z-stacked confocal slices of viral infection utilizing 3D volume-rendering software to determine if AAV2 virions colocalized with wild-type CFTR (Fig. 5B, panel ii, red) or ΔF508-CFTR (panel iii, red) in BHK-21 cells. We did not detect any obvious colocalization between capsids and wild-type CFTR or ΔF508-CFTR, although we could not rule out the possibility that components of their trafficking pathways converge upstream of their degradation at a separate subcellular location.

### Localization of AAV capsids compared to perinuclear cellular markers

**Figure 6:**
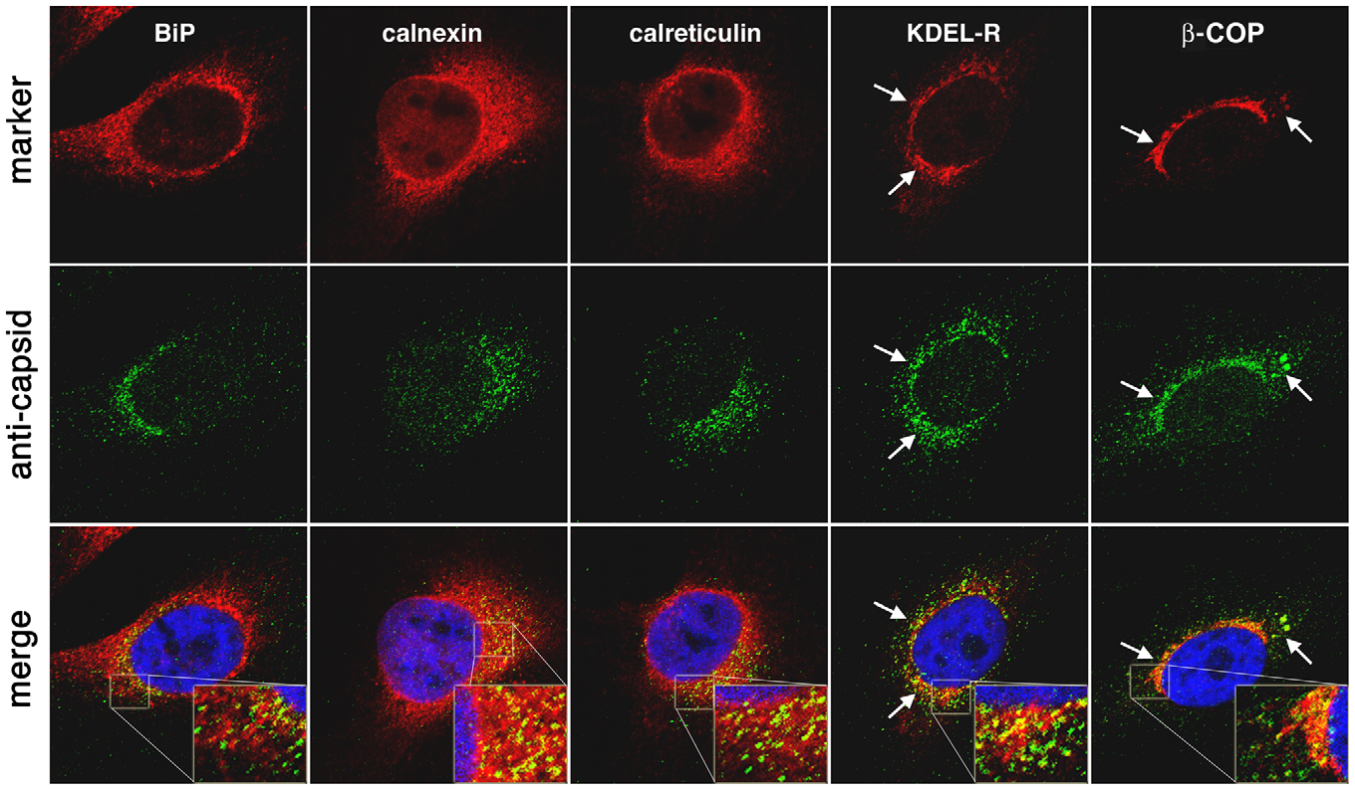
AAV capsids traffic to locations near Golgi/ER transport components. AAV2 virions (10,000 vg/cell) were administered to HeLa cells for 16 hr and prepped for confocal immunofluorescence with antibodies directed toward cellular markers (red) for ER-resident chaperones (BiP, calnexin, calreticulin), Golgi sorting components (KDEL-R,β-COP), or AAV2 capsids (green). Nuclei (blue) were stained with DAPI. White arrows indicate partial colocalization with KDEL-R and β-COP, yet it is difficult to assess whether colocalization occurs with ER markers.

Two major cellular compartments arranged in a perinuclear fashion are the Golgi apparatus and ER, which are oriented towards the MTOC. AAV serotypes have previously been reported to associate with the Golgi during infection, although the significance of this association is not yet understood, [23], [38]. To date, AAV has not been shown to enter the ER, but other viruses such as SV40 will engage retrograde transport pathways from endocytic compartments to the ER, undergo disulfide processing in the ER, and then translocate into the cytosol in order for subsequent steps in infection to proceed [39], [40], [41]. It is not clear if AAV requires processing in either the ER or Golgi during infection, but a preponderance of capsids are found to accumulate perinuclearly. With this in mind, we asked whether we could detect capsids in the secretory pathway near well characterized markers of the ER, Golgi, and factors that route cargo between these compartments. We have previously established that perinuclear accumulation of capsids occurs early after infection and peaks in intensity around 16 hr post inoculation [42] in HeLa cells. Therefore, we inoculated HeLa cells with AAV2 and prepped samples for confocal immunofluorescence 16 hr later with antibodies directed toward cellular markers (red) for ER-resident chaperones (BiP, calnexin, calreticulin), Golgi sorting components (β-COP, KDEL-R), or AAV2 capsids (green) (Fig. 6). No obvious colocalization was found between AAV capsids and traditional markers for the ER, BiP/Grp78, calnexin, or calreticulin [43]. Although fluorescent signal corresponding to capsids emanated from the same area of the cell as BiP, calnexin, or calreticulin, these signals predominantly did not overlay with one another. Moderate colocalization was detected between AAV2 and KDEL-R, a receptor that recognizes lysine-aspartate-glutamate-arginine (KDEL) lumenal signal sequences at the C-terminus of proteins [44], [45], [46]. KDEL-R serves as an ER-retention device for misfolded proteins and transitions between the Golgi and the ER [44]. We detected significant colocalization with β-COP (Fig. 6), a component of the coatomer complex that is typically associated with COPI transport in retrograde direction from the Golgi to the ER [47]. While we and others have previously demonstrated that rAAV capsids colocalize with markers for Golgi cisternae [23], [38], [48], such as giantin, the data here indicate that capsids may also route through other structures in the post-ER secretory system. KDEL-R and β-COP are also known to sort proteins through the ER-Golgi intermediate compartment, but we did not observe significant capsid colocalization with a marker for this compartment, ERGIC-53 (data not shown), which is consistent with reports stating that ERGIC-53 is more well-represented in the ER than either KDEL-R or β-COP [49]. While we did not find substantial evidence of AAV2 colocalization with the ER, the partial colocalization between AAV2 and KDEL-R or β-COP (Fig. 6, white arrows) supports the notion that an AAV2 capsid population traffics through the Golgi apparatus and might be further routed through the secretory pathway in a retrograde direction.

### siRNA-mediated knockdown of secretory pathway components influences AAV infection efficiency

**Figure 7:**
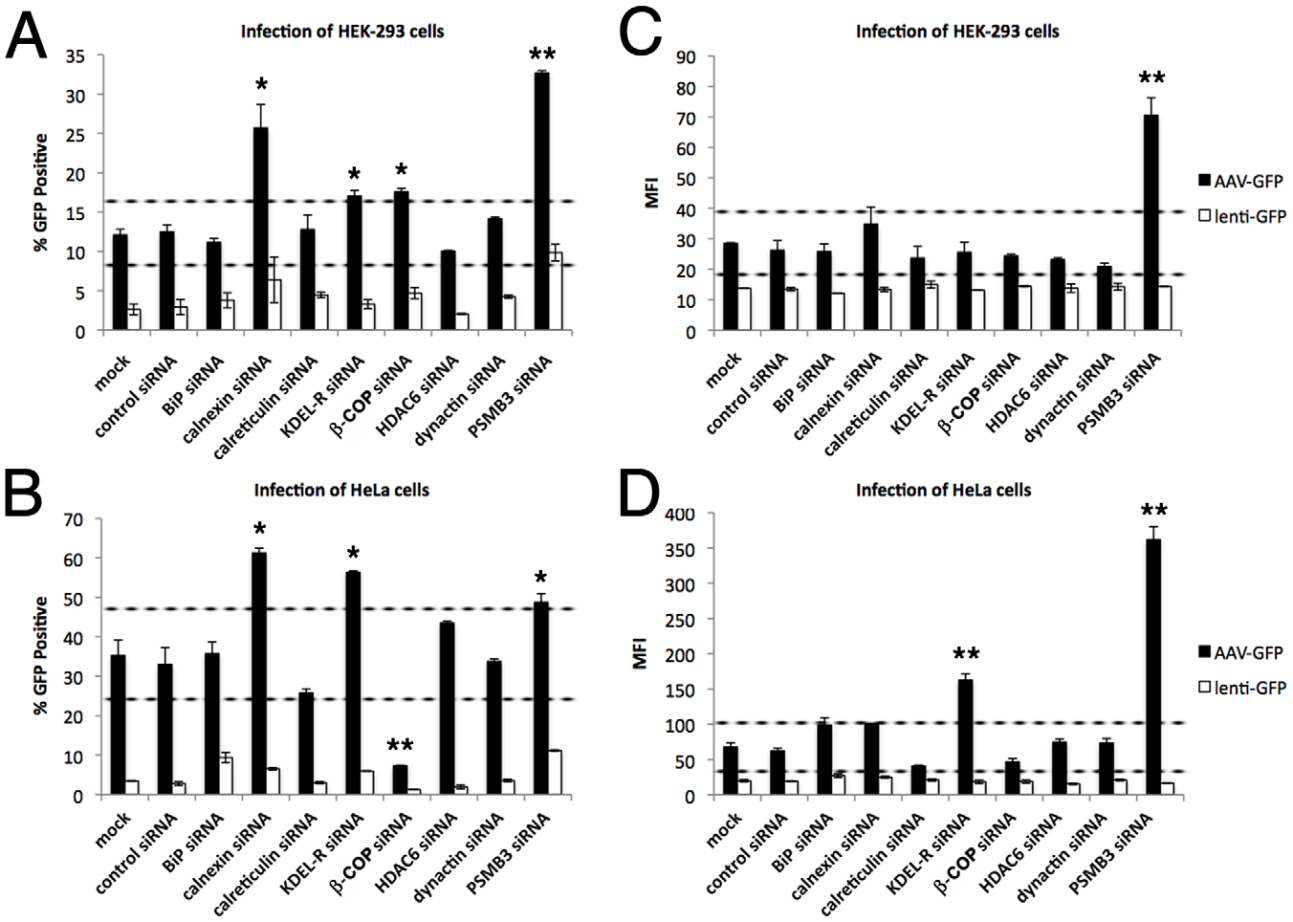
Knockdown of secretory pathway components or proteins involved in CFTR processing impacts AAV infection. Transduction efficiency of AAV following siRNA-mediated knockdown of proteins in HEK-293 cells (A & C) or HeLa cells (B & D). A margin of error for nonsignificant perturbations of infection (roughly ±30% relative to controls) is indicated by dashed lines. Cells were mock transfected or transfected with non-targeting control siRNA, or siRNA targeting BiP, calnexin, calreticulin, KDEL-R, β-COP, HDAC6, dynactin, or PSMB3. Between 36 and 48 hr after transfection, cells were infected with AAV2-GFP (200 vg/cell, HEK-293; 500 vg/cell HeLa) and fixed for flow cytometry 24 hr post-infection. Data is reported as the percentage of cells infected (% GFP positive, A & B) or mean fluorescence intensity (MFI, C & D) from cells that are positive. Differences in cell-type specificity are apparent with β-COP, but ultimately these results suggest AAV infection can be influenced by components of the secretory pathway and the proteasome. Cells infected with lenti-GFP as a control, do not display significant differences in MFI (C & D, black columns), ruling out aberrant effects on reporter gene expression. Error bars represent standard deviation and graphs are representative data sets of at least three independent experiments. Samples that were statistically different from controls are marked (*p<0.05; **p<0.01).

Using microscopy methods alone, it is difficult to determine whether sorting of capsids through the secretory pathway is a necessary step during infection. Several studies including this one, have used physical or pharmacological stressors that perturb secretory system traffic to enhance AAV transduction (supplementary Fig. S2), but these agents have a multitude of other cellular effects [28], [50], [51], [52]. To test if knockdown of specific components could positively or negatively impact AAV infection, we employed siRNA-mediated knockdown of proteins involved in ER quality control (BiP, calnexin, calreticulin), proteins involved in retrograde transport (KDEL-R, β-COP), proteins required for aggresomes formation and autophagy (HDAC6, dynactin), and proteasome subunit beta type-3 (PSMB3). HEK-293 cells (Fig. 7, A & C) or HeLa cells (Fig. 7, B & D) were mock transfected or transfected with non-targeted control siRNA, or siRNAs directed toward the aforementioned targets. Specific knockdown for each target was verified two days after transfection by western analysis of protein levels or by quantifying cellular mRNA levels compared to controls (supplementary Fig. S3). After knockdown, cells were replated at equal densities, inoculated with AAV2-GFP, and fixed for flow cytometry 24 hr post-inoculation. Under these conditions, no significant difference in either percentage of cells infected (% GFP positive, Fig. 7A & B) or mean fluorescence intensity (MFI, C & D) was observed between samples treated with mock conditions, control siRNA, BiP siRNA, calreticulin siRNA, HDAC6 siRNA, or dynactin siRNA. Notably, siRNA-mediated knockdown of calnexin increased the percentage of cells infected with AAV-GFP in both HEK-293 and HeLa cells. Knockdown of KDEL-R increased the number of GFP-positive HeLa cells and augmented expression intensity (Fig. 7, B & D), and we found a similar effect on the number of cells transduced in HEK-293 samples (Fig. 7A). Interestingly, we observed cell-type specific effects from knockdown of β-COP, which resulted in a modest increase of AAV transduction in HEK-293 cells, but in contrast, led to a significant decrease (more than 5-fold) in the percentage of transduced HeLa cells. With respect to the negative effect of β-COP siRNA on AAV transduction in HeLa cells, this outcome may simply be due to a reduction in cell viability or global disruption of secretory pathway traffic, as previously reported [53]. Cells transduced with a lenti-GFP control vector, did not display significant differences in MFIs from GFP-positive cells under these conditions (Fig. 7, C & D, empty columns), ruling out aberrant effects on reporter gene expression.

Additionally, knockdown of PSMB3 significantly increased the percentage of GFP-positive cells transduced by AAV, and had a dramatic effect on MFI for both HEK-293 and HeLa cell types. Since PSMB3 is a part of the 20S core of the proteasome, this finding is consistent with the notion that the ubiquitin/proteasome system plays a role to restrict AAV infection. As functional evidence for this effect, siRNA targeting PSMB3, but not other secretory pathway components, reduced catalytic activity of the proteasome (supplementary Fig. S4). We also noted that knockdown of PSMB3 increased the number of GFP-positive cells transduced by lenti-GFP, in accord with previous lentiviral studies with proteasome inhibitors [54], [55], which supports the hypothesis that the proteasome system is involved in restricting entry or processing of viruses from more than one class.

The siRNA-mediated effects on AAV transduction were not due to an indirect effect on virus uptake, as similar numbers of vector genomes were present in cells after infection as measured by qPCR (supplementary Fig. S5). Additionally, the same modulated patterns of transduction in siRNA-treated HEK-293 and HeLa cells were found for self-complementary AAV2 (supplementary Fig. S6), ruling out the possibility that increased transduction resulted from increased efficiency of second-strand synthesis. We have demonstrated that infection efficiency can be significantly affected by ablating proteins involved in sorting or degradation: calnexin, KDEL-R, β-COP, or PSMB3. Results from our studies suggest that a population of AAV virions is routed through the post-ER secretory pathway during entry, and these virions experience an infectious advantage when this pathway is compromised.

## Discussion

These studies describe how subcellular stress increases susceptibility to infection with the parvovirus, AAV, in cellular models of CF. We report that AAV infection efficiency is significantly higher in HAE when cultures are stressed by exposure to SMM (Fig. 1), an inflammatory stimulant derived from diseased lungs that models physiological conditions present in CF airways. Granting that SMM is a pleiotropic model of subcellular stress, we demonstrate independently that expression of misfolded ΔF508-CFTR is sufficient to increase infection efficiency by 5- to 10-fold (Figs. 3 & 4). A similar opportunistic phenomenon also occurs with the human respiratory pathogens, Ad and RSV (Fig. 2), which are known to have a disproportionately greater impact on CF patients than on normal individuals [56], [57]. Although it remains to be seen if the same mechanisms are responsible for enhancing virus infection in all cases, effects related to protein misfolding were primary factors involved in the increased susceptibility to AAV. Rescuing misfolded ΔF508-CFTR with low temperature conditioning blocked the elevation in rAAV2 infection efficiency. Additionally, using a panel of mutant CFTR proteins we show this phenomenon is common to other misfolded proteins and not related to loss of CFTR activity, since the G551D-CFTR mutant did not confer increased susceptibility to AAV infection. While we did not find direct colocalization between AAV and CFTR proteins, we were able to detect virus capsids in regions of the secretory pathway that correspond to retrograde trafficking out of the Golgi apparatus. Furthermore, we have demonstrated that infection efficiency can be modulated by siRNA-mediated knockdown of components in the secretory pathway or the proteasome. With this work we have provided evidence that factors in the secretory pathway are enlisted to process misfolded proteins and protect against infection.

Manipulation and subversion of stress responses is a common thread in virus replication. Hepatitis B virus is known to activate an ER stress response in order to optimize production of its surface proteins [58]. Japanese encephalitis virus infection similarly triggers ER stress and activates an unfolded protein response, thus promoting apoptosis and progeny dissemination [59]. Other viruses, such as human cytomegalovirus and herpes viruses, have evolved mechanisms that activate chaperone upregulation and expand the ER, while controlling unwanted consequences of eIF2α phosphorylation, which would normally inhibit protein translation [60], [61]. In our studies, the AAV reporter virus has been stripped of its wild-type viral genome and does not replicate. Taken together with the fact that no difference was observed in ΔF508-CFTR-expressing cells with respect to virus binding, and the effect on transduction was not observed in promoter transgene cassette transfection studies (supplementary Fig. S1), our focus became more narrowed toward studying how exploitation of subcellular stress may occur during virus entry.

A variety of drug treatments and physical stressors are known to increase infection efficiency of AAV through effects on capsid processing or genome amplification [28], [50], [51], [52]. It should be noted that AAV capsids are trafficked to the MTOC early during infection (supplementary Fig. S2A), a location where misfolded proteins are targeted for degradation [62]. Typically, select cytoplasmic proteins are marked for degradation by ubiquitin, a signaling hallmark that can direct either a change in substrate trafficking or promote substrate degradation via the proteasome. Previous research has demonstrated AAV serotypes -2 and -5 can be ubiquitinated during in vitro reactions and during infection [63]. Likewise, pharmacological inhibition of the proteasome results in a potent increase in AAV infection efficiency in a cell- and serotype-specific manner [28], [64], although the mechanisms responsible are not completely understood. This prompts the question of whether enhanced infection of ΔF508-CFTR cells is due to proteasome inhibition. Surprisingly, we have found that proteasomes function at relatively the same rate in ΔF508-CFTR-expressing cells as in control cells and CFTR-expressing cells, according to a luciferase assay of proteasome activity (supplementary Fig. S7A). Yet, supporting our hypothesis, the use of proteasome inhibitors in control cells or cells expressing wild type CFTR increases AAV transduction to just over that of untreated cells expressing ΔF508-CFTR (see supplementary Fig. S7B). Treating ΔF508 cells with proteasome inhibitors results in a partial increase in transduction that is not as effective as in control cells (a three-fold increase compared to 10-fold in controls). These data point to the proteasomal pathway as having a significant impact on viral transduction in this setting. While it is tempting to speculate that dysfunction of the ubiquitin/proteasome system is a primary factor leading to AAV's ability to exploit the stressed subcellular environment, our siRNA results (Fig. 7) suggest that other changes in the secretory pathway are additionally at play. Together, our findings suggest the increase in susceptibility to AAV infection in ΔF508 cells is not strictly due to a direct inhibition of the proteasome, but could also be due to saturation or sequestration of pathway components upstream of catalytic action.

Maintaining homeostasis in the secretory pathway is paramount for proper folding or disposal of misfolded proteins. Throughout its biogenesis and degradation, ΔF508-CFTR interacts with a multitude of proteins in the cell [65] and may influence the effectiveness of quality control machinery in the post-ER secretory pathway. One hypothesis is that during stressed conditions, expression of ΔF508-CFTR or other misfolded proteins may saturate or sequester proteins with chaperone and quality control function. AAV infection may be enhanced if these proteins would otherwise impede virus trafficking or processing. Indeed, we have identified overlapping systems that process ΔF508-CFTR and influence AAV. Proteins known to play a role in ΔF508-CFTR processing include the chaperones calnexin, Hsp90, and the Hsc-Hsp40/Hsp70 complexes [4]. In one report, Hsp90 has been shown to inhibit AAV infection [66], and similarly, we see that the Hsp90 inhibitor radicicol potently increases transduction (supplementary Fig. S1B), as does siRNA-mediated knockdown of calnexin (Fig. 7). Although siRNA-mediated knockdown of calnexin increases AAV transduction in HEK-293 and HeLa cells, this does not necessitate an interaction between calnexin and AAV capsids. Loss of calnexin has been shown to be associated with an increased constitutively active unfolded protein response [67]. While many viruses have been shown to manipulate the unfolded protein response during infection, it is unclear whether this influences AAV transduction. It is noteworthy to mention that activating or inactivating a major axis of the unfolded protein response through IRE1/XPB-1, which is linked to ER expansion and Ca^2+^-mediated inflammation in human airway epithelium [30], did not impact rAAV2 transduction in a human bronchial epithelial cell line (supplementary Fig. S8). However, we cannot rule out that other arms of the unfolded protein response (ATF6 or PERK pathways) could be involved, or that factors in addition to, and/or superseding an unfolded protein response might be engaged to mediate increased AAV transduction during subcellular stress.

Interestingly, siRNA-mediated knockdown of HDAC6 or dynactin did not significantly impact AAV infection under the conditions tested. These proteins are putatively required for microtubule-based transport of misfolded proteins like ΔF508-CFTR to aggresomes and autophagosomes [68], [69]. In agreement with these siRNA studies, we did not find AAV capsids to colocalize with cellular markers for aggresomes or autophagosomes in HeLa cells (vimentin, atg5, atg7, LC3; data not shown). However, we cannot conclude that AAV infection is unaffected by autophagic processes, especially since we observe 3-methyladenine, an inhibitor of autophagy to increase AAV-mediated gene delivery (supplementary Fig. S1B).

The most significant siRNA-mediated effect on AAV infection in these studies was found with knockdown of PSMB3 (Fig. 7), a proteasome subunit that has been reported to interact with ΔF508-CFTR, but not wild-type CFTR [65]. PSMB3 knockdown increased the number of GFP-positive cells, but more impressively augmented expression intensity in both HEK-293 and HeLa cells. Knocking down KDEL-R or β-COP also modulates AAV infection efficiency. It is not clear what role these proteins may play in AAV trafficking, but classically KDEL-R and β-COP are retrograde transport proteins, which serve as ER-retention devices for misfolded proteins that transition between the Golgi, ER-intermediate compartment, and the ER [44]. KDEL-R was recently identified as a potential AAV capsid binding partner in a yeast two-hybrid screen [70]. Capsid mutagenesis studies may in the future shed light on how AAV is recognized by cellular sensors in the post-ER secretory pathway, and potentially elucidate how these factors could restrict viral trafficking. Additionally, we'd like to note that the endocytic and secretory systems in polarized cells are quite different from that in non-polarized cells. In polarized airway epithelium, the nucleus is positioned near the basolateral membrane, whereas the ER and Golgi are oriented sub-apically at the minus end of microtubules. It is currently unclear whether AAV traffics differently in polarized and non-polarized cells, and how this would pertain to viral exploitation of subcellular stress. Considering that viral exploitation of stress may not be related to a trafficking block occurring at a specific subcellular location, an attractive hypothesis is that altered capsid processing underlies enhanced transduction (such as ubiquitination, phosphorylation, proteolytic processing, etcetera).

The facets of subcellular stress discussed here, such as expansion of the ER, changes in the secretory pathway, activation of the unfolded protein response, and dysfunction of the ubiquitin/proteasome system, will likely have a profound effect on a cell's ability to combat infection. In this report we have illustrated that the consequences of these subcellular stresses, elicited by exposure to SMM or the presence of misfolded proteins, can render cells more susceptible to AAV in cellular models of CF. Irrespective of whether all of these effects contribute to pathogenesis in diseased airways, it is clear they are associated with numerous misfolded protein diseases [71]. In one instance, it has been shown that adaptation to constitutive expression of misfolded surfactant protein C, led to increased susceptibility to RSV infection in vitro [72], corresponding with enhanced cytotoxicity. It will be of great interest to uncover if exploitation of subcellular stress by pathogens is a generalized phenomenon that occurs in prion diseases, Parkinson's disease (α-synuclein), amyotrophic lateral sclerosis (superoxide dismutase-1), hereditary emphysema (α-1-antitrypsin), familial neurohypophyseal diabetes insipidus (arginine vasopressin), or many other disorders that stem from misfolded or unfolded proteins [73]. Even more striking, would be to discover that there is a viral component to the progression of some of these diseases. If the presence of misfolded proteins or other subcellular insult, might render certain cells more susceptible to infection, this parameter should also hold true for viral-mediated therapies. These may be important angles to consider as future research shapes new ways to understand, prevent, and provide treatment for cells under threat from internal or external stressors.

## Materials and Methods

### Cell culture

HeLa cells were maintained at 37°C and 5% CO2 in Dulbecco's modified Eagle's medium (DMEM) that was supplemented with 10% heat-inactivated fetal calf serum, 100 U/ml penicillin, and 100 g/ml streptomycin. BHK-21 cells were grown at 37°C and 5% CO2 in Dulbecco's modified Eagle's medium (DMEM) and Ham's F12 medium (50∶50) that was supplemented with 5% heat-inactivated fetal calf serum, 100 U/ml penicillin, and 100 g/ml streptomycin. BHK-21 clones expressing wild-type CFTR, CFTR-ΔF508, CFTR-G551D, CFTR-D572N, CFTR-1410X, wild-type MRP1, or MRP1-ΔF728 were maintained in selection medium containing methotrexate (250 µg/ml). Presence or absence of methotrexate was not found to influence viral transduction (data not shown), and selection was continued throughout the course of infection.

### Generation of ciliated HAE in vitro

Human tracheobronchial epithelial cells were isolated fresh from excess normal surgical specimens from lung transplantation by the University of North Carolina Cystic Fibrosis Center Tissue Culture Core under University of North Carolina institutional review board–approved protocols. As previously described, primary cells derived from single patient sources were expanded in tissue culture to generate passage 1 cells, which were plated at a density of 250,000 cells/cm^2^ on permeable membrane support (Millicells, 12 mm diameter, Millipore, Corning, NY), and maintained in a specialty media [74]. Cells were differentiated under air–liquid interface conditions and the cultures were considered mature after 4–8 weeks when found to be significantly ciliated (>50% ciliated cells). Experiments were only performed with fully differentiated HAE cultures.

### Virus production

Recombinant AAV or lenti-GFP was produced as described before in HEK-293 cells [75], [76]. RSV packaging GFP was generated as previously described in HEp-2 cells [77]. Ad type 5 packaging GFP was obtained from the University of North Carolina Vector Core Facility. Briefly, for production of AAV, polyethylenimine (linear molecular weight, ∼25,000) was used for triple transfection of the pXR2 cap and rep plasmid, the pXX6-80 helper plasmid, and pTR-Luciferase or TR-GFP reporter plasmids containing either luciferase or enhanced green fluorescent protein (GFP) transgenes flanked by inverted terminal repeats. At 60 hr post-transfection cells were harvested and virus purified by cesium chloride gradient density centrifugation for 5 hr at 65,000 rpm or overnight at 55,000 rpm in an NVT65 rotor (Beckman). Virus was dialyzed into 1× phosphate-buffered saline (PBS) to remove small molecular weight contaminants from fractions containing peak viral titers, which were identified by dot blot hybridization. Titers were calculated by qPCR according to established procedures [42] using a LightCycler 480 using Sybr green (Roche) and primers designed against *firefly* Luciferase (TR-CBA-Luc) or enhanced green fluorescent protein (TR-CMV-GFP) transgene.

### Collection of SMM and transduction of HAE cultures

Lumenal mucopurulent material from excised human CF lungs was harvested at the North Carolina Cystic Fibrosis Center as previously described [8], [9], [30]. To ensure a homogenous stimulus, SMM was pooled from several patients. For treatment prior to infection, SMM (20 µl) was added to the apical surface of fully differentiated HAE cultures in Millicells for 24 hr. Prior to virus administration, SMM-treated wells and non-treated control wells were washed two times with PBS. An AAV variant (HAE-2) previously isolated from directed evolution in HAE cultures [31] was used in these studies. Approximately 5×10^10^ particles of HAE-2 packaging either GFP or Luc were applied to the apical surface of HAE cultures in PBS for 4 hr. Virus was washed from the apical surface and GFP or Luc expression was assayed 1 week post-infection.

### Luciferase assay of transduction

At least 4 hr prior to infection, HeLa cells or BHK-21 cells were plated in 24 well plates at a density of 10^5^ cells/well. 24 hr after inoculation with AAV at the indicated vg/cell numbers, luciferase activity was measured in accordance with the manufacturer's instructions (Promega) and with a Wallac1420 Victor2 automated plate reader. Error bars represent standard deviations from samples scored in triplicate. Graphs are representative data sets from at least three independent assays.

### GFP flow cytometry analysis of transduction or transfection

BHK-21 cells were prepared for infection as stated above and plated in 24 well plates at a density of 10^5^ cells/well. After infection at the indicated MOI, at 24 or 48 hr cells were trypsinized (100 µl/well), neutralized with growth media (300 µl) spun down for 5 min at 500 g, and the cell pellets were resuspended in 500 µl of 1% paraformaldehyde (prepared fresh in PBS). At least 50,000 cells were counted by flow cytometry for each sample, with at least three separate samples recorded for each condition. Graphs are representative data sets from one of three independent experiments.

### Western blotting

CFTR and MRP1 protein samples were analyzed as previously described [35]. Briefly, cells were lysed and subjected to SDS-PAGE gel electrophoresis on 6% polyacrylamide gels. Proteins were transferred to nitrocellulose membranes and blots were probed with anti-CFTR monoclonal 596 or monoclonal anti-MRP1 antibody 897.2 and visualized by enhanced chemiluminescence detection (General Bioscience).

### Quantifying genomes by PCR

DNA was isolated from cell samples using DNeasy columns (Qiagen) according to the manufacturers protocol. Quantitative PCR was performed on a LightCycler 480 using Sybr green (Roche) and primers designed against the *firefly* Luciferase transgene: (fwd) 5′ AAA AGC ACT CTG ATT GAC AAA TAC 3′, and (rev) 5′ CCT TCG CTT CAA AAA ATG GAA C 3′. Conditions used for the reaction were as follows: 1 cycle at 95°C for 10 min, 45 cycles at 95°C for 10 sec, 62°C for 10 sec, 72°C for 10 sec, 1 cycle at 95°C for 30 sec, 65°C for 1 min, and 99°C for acquisition.

### Transfection of BHK-21 cells with reporter plasmids

BHK-21 control cells, CFTR cells, or ΔF508 cells were plated at a density of 10^5^ cells/well and 4 hr later were transfected with either TR-CBA-Luc or TR-CMV-GFP reporter plasmids using PEI. For each well 500 ng DNA was mixed with 5 µl PEI (1 mg/ml) in 100 µl of serum-free DMEM and incubated at room temp for 10 min before adding the mixture to cell medium. 24 hr after transfection, cells were harvested for luciferase assays or flow cytometry analysis of GFP expression as indicated below.

### Confocal immunofluorescence microscopy

Similar to what we have previously described [42], [78], HeLa cells or BHK-21 cells (5×10^4^ cell/well) were plated on poly-L-lysine-coated 12 mm glass coverslips (#1.5) 4 hr before infection. AAV2 virions were added to cell media at designated vector genome amounts. No virus was added to control wells. After infection, cells were washed three times with PBS and then fixed with 2% paraformaldehyde for 15 min at room temperature. Cells were then permeabilized with 0.1% Triton X-100 in PBS for 5 min at room temperature, washed four times with PBS, and then blocked with immunofluorescence buffer (IFB) (20 mM Tris, pH 7.5, 137 mM NaCl, 3 mM KCl, 1.5 mM MgCl_2_, 5 mg/ml bovine serum albumin, 0.05% Tween) for 30 min at room temperature. The cells were incubated with primary antibody to detect intact capsids [(monoclonal A20 (1∶10)], or cellular markers (anti-γ-tubulin (Abcam Ab16504 1∶250), anti-BiP (Abcam Ab21685 1∶1000), anti-calnexin (Abcam Ab 13504 1∶1000), anti-calreticulin (Abcam Ab2907 1∶1000), anti-KDEL-R (Santa Cruz Biotechnology sc-33806 1∶50), anti-β-COP (Abcam Ab2899 1∶1000), or anti-CFTR-570 (1∶500)] diluted in IFB, for 1 hr at 37°C or overnight at 4°C. The cells were then incubated in secondary antibody, diluted 1∶5000 in IFB (anti-mouse Alexa Fluor 488; or anti-rabbit Alexa Fluor 568, anti-mouse IgG3 Alexa Fluor 488, or anti-rabbit IgG1a Alexa Fluor 568, Molecular Probes), for 1 hr at 37°C. After six washes in PBS, coverslips were mounted cell side down on glass slides with mounting medium (Prolong antifade Gold with DAPI [4′,6′-diamidino-2-phenylindole]; Molecular Probes). Images were captured on a Leica SP2 AOBS upright laser scanning confocal microscope and processed for brightness or contrast using Photoshop CS4 (Adobe). Confocal Z-stack sections of BHK-21 cells for immunofluorescence localization of AAV2 capsids and CFTR or ΔF508-CFTR protein were processed and rendered in 3-dimensions using Volocity software (Improvision).

### siRNA transfection

Specific knockdown of secretory pathway components or factors that process misfolded proteins was obtained by double transfection of siRNA (Qiagen, Flexitube) using Hiperfect (Qiagen) according to the manufacturer's protocol for fast-forward transfection of adherent cells. Briefly, 200,000 HEK-293 cells or 70,000 HeLa cells were plated into 24 well plates and mixed immediately with pre-incubated DMEM, transfection reagent, and siRNA at a final concentration of 10 nM. After verifying knockdown, approximately two days post-transfection cells were trypsinized and replated at equal densities for transduction assays using either AAV2-GFP or HIV-GFP. Knockdown was verified by analyzing protein levels by western blot or by quantifying mRNA from transfected cells compared to controls by using SuperScript III CellsDirect cDNA Synthesis Kit (Invitrogen) according to the manufacturer's instructions, and subsequently testing samples by qPCR. Target accession numbers were as follows: BiP - NM_005347; calnexin - NM_001746; calreticulin - NM_004343; KDEL-R3 - NM_006855; β-COP - NM_001144061; HDAC6 - NM_006044; dynactin4 - NM_016221; PSMB3 - NM_002795. Catalogue numbers for the paired siRNAs were as follows: BiP –SI02780554, SI02781016; calnexin – SI02663367, SI02757300; calreticulin – SI02654589, SI02777096; KDEL-R3 –SI02638181, SI03100580; β-COP – SI00299887, SI04141438; HDAC6 - SI02663808, SI02757769; dynactin4 – SI00360444, SI00360437; PSMB3 – SI02654239, SI02653882.

### Statistical analysis

For studies involving quantification of luciferase or GFP expression a one-way analysis of variance (ANOVA) was used to determine if a statistical difference existed between mean values. If any of the means differed, a Tukey HSD test was performed to determine p-values for sample means that differed from control BHK-21 cells and BHK-21 cells expressing wild-type CFTR. A p-value≤0.05 was considered significant and marked in the figures as (*). A p-value≤0.01 is marked (**). All data were analyzed using Excel software (Microsoft).

### Accession numbers

For information on cystic fibrosis (CF) please see Online Mendelian Inheritance in Man database (MIM) #219700. The CFTR protein can be referenced through GeneID: 1080, and CFTR mutations in association with disease through MIM: 602421. The viruses discussed in the manuscript text have the following accession numbers: adeno associated virus - Refseq: NC_001401, Taxonomy ID: 10804; adenovirus - Refseq: AC_000008, Taxonomy ID: 28285; respiratory syncytial virus - Taxonomy ID: 11250. Proteins targeted for the siRNA studies included the following: BiP - GeneID: 3309; calnexin - GeneID: 821; calreticulin - GeneID: 811; KDEL-R - GeneID: 11015; β-COP - GeneID: 1315; Dynactin - GeneID: 479326; HDAC6 - GeneID: 10013; and PSMB3 - GeneID: 5691.

## Supporting Information

Figure S1 Exploitation during infection likely occurs between virus binding and gene expression. A. Cell surface binding of AAV2. Equal numbers of control wt BHK-21 cells (purple diamonds), cells expressing CFTR (red squares), or ΔF508-CFTR (blue triangles) seeded in 24 well plates were cooled to 4°C to inhibit endocytosis. AAV2 was applied (10,000 vg/cell) for the indicated times, and the DNA was harvested for qPCR quantification of vector genomes. Error bars represent standard deviations from three independent samples. B. Analysis of expression levels after transfection of reporter plasmids by flow cytometry indicating mean fluorescence intensity (MFI) of GFP-positive cells 24 hr after transfection of TR-eGFP in control cells, cells expressing CFTR, or ΔF508-CFTR C. Luciferase assay of reporter expression in control cells, CFTR-expressing cells, or ΔF508-CFTR expressing cells, 24 hr after transfection with a TR-Luc plasmid. Error bars represent standard deviations from three samples.
(TIF)

Figure S2 Subcellular stress pathways in trafficking and transduction. A. Confocal immunofluorescence microscopy depicts AAV2 capsids (i, green) trafficking near the microtubule organizing center (ii - γ-tubulin, red) at 4 hr post-infection in HeLa cells. B. Luciferase assay of transduction showing several treatments that influence misfolded protein processing positively impact AAV2 transduction. AAV2 capsids (10,000 vg/cell) were administered concurrently with either vehicle controls (DMSO or EtOH), or ALLN (40 µM), MG132 (2 µM), Radicicol (10 µM), 3-methyladenine (10 mM), or administered directly after treating with L-canavanine (10 mM, 12 hr), or during heat shock (4 hr at 42.5°C). DMSO and EtOH did not significantly influence expression. The proteasome inhibitors ALLN and MG132 increased transduction between 30 and 40-fold. The Hsp90 inhibitor, radicicol, known to perturb chaperone activity and induce a heatshock-like response [79], increased transduction by roughly 100-fold. 3-methyladenine, an inhibitor of autophagy [80], [81], augmented transduction by at least 20-fold. L-canavanine, an amino acid analogue of arginine, induces widespread protein misfolding [82] and increased AAV transduction 5- to 10-fold. Heat shock also increased transduction as previously documented [51]. Luciferase activity was measured 24 hr post-infection.
(TIF)

Figure S3 siRNA-mediated knockdown of secretory pathway components or proteins involved in CFTR processing. A. Representative Western blots demonstrating levels of target protein reduction compared to GAPDH or β-actin controls after siRNA transfection (∼48 hr), corresponding to time of infection. B. For those targets where a reliable antibody was not available for Western blot (KDEL-R and PSMB3), we quantified levels of mRNA by RT-qPCR, normalized to mRNA for GAPDH. Transfections for mock conditions (transfection reagent alone), control (non-targeted siRNA), or two target siRNAs were performed in HEK-293 and HeLa cells according to procedures described in Materials and Methods.
(TIF)

Figure S4 Chymotrypsin-like proteasome activity in cells transfected with siRNA. Two days following transfection with non-targeted control or indicated targeted siRNAs, HEK-293 (A) or HeLa cells (B) were split to 20,000 cells/well in 96 well plates and tested for proteasome activity using a cell-based luciferase assay (Promega G8660) according to the manufacturer's instructions. Data shown are representative of two experiments with standard deviations from three independent samples.
(TIF)

Figure S5 Quantification of viral uptake in cells transfected with siRNA. Between 36 and 48 hr following transfection with non-targeted control or indicated targeted siRNAs, HEK-293 (A) or HeLa cells (B) were inoculated with rAAV2 (5,000 vg/cell or 10,000 vg/cell, respectively) for 24 hr. Total DNA was harvested from cells by DNeasy preparation according to procedures in Materials and Methods and viral genomes were quantified by qPCR which were normalized to GAPDH levels. Data shown represent averages with standard deviations from two independent experiments.
(TIF)

Figure S6 Transduction efficiency of self-complementary rAAV2 in cells transfected with siRNA. Transduction efficiency of self-complementary AAV2 (scAAV2) following siRNA-mediated knockdown of proteins in HEK-293 cells (A) or HeLa cells (B). A margin of error for nonsignificant perturbations of infection (roughly ±30% relative to controls) is indicated by dashed lines. Cells were mock transfected or transfected with non-targeting control siRNA, or siRNA targeting BiP, calnexin, calreticulin, KDEL-R, β-COP, HDAC6, dynactin, or PSMB3. Between 36 and 48 hr after transfection, cells were infected with scAAV2-GFP (50 vg/cell, HEK-293; 100 vg/cell, HeLa) and fixed for flow cytometry 24 hr post-infection. Data is reported as the percentage of cells infected times mean fluorescence intensity. Data is representative of two independent experiments with error bars indicating standard deviation. Samples that were statistically different from controls are marked (*p<0.05).
(TIF)

Figure S7 Influence of the ubiquitin/proteasome system on AAV infection in models of CF. A. Luciferase assay of proteasome activity in BHK-21 cells with or without proteasome inhibitor (MG132, 2 µM). B. Luciferase assay of AAV2 transduction in HEK-293 cells with or without proteasome inhibitor. AAV2 capsids (10,000 vg/cell) were administered to equal numbers of control, CFTR, or ΔF508 cells in 24 well plates and scored for transduction 24 hr after inoculation. Proteasome inhibition potentiates infection efficiency in each cell type, but is less effective in ΔF508 cells.
(TIF)

Figure S8 Influence of XBP-1 splicing and the unfolded protein response on rAAV2 transduction. Transformed human bronchial epithelial cells (16HBE14o-, control), or 16HBE14o- cells stably expressing dominant negative XBP-1 (DN-XBP-1), or expressing constitutively active XBP-1 [spliced XBP-1; (Lee, A. H., Iwakoshi, N. N., and Glimcher, L. H. (2003.) Mol. Cell. Biol. 23 7448–7459)], were inoculated with rAAV2 at the indicated particle numbers. After 24 hr a luciferase assay of transduction was performed to measure efficiency of AAV2-mediated gene delivery. Error bars represent standard deviations from four independent samples.
(TIF)
