# Supplementary figures and images for: AAV Exploits Subcellular Stress Associated with Inflammation, Endoplasmic Reticulum Expansion, and Misfolded Proteins in Models of Cystic Fibrosis
Source: PLoS Pathog. 2011 May 19;7(5):e1002053. doi: 10.1371/journal.ppat.1002053 (PMC3098238; doi:10.1371/journal.ppat.1002053)

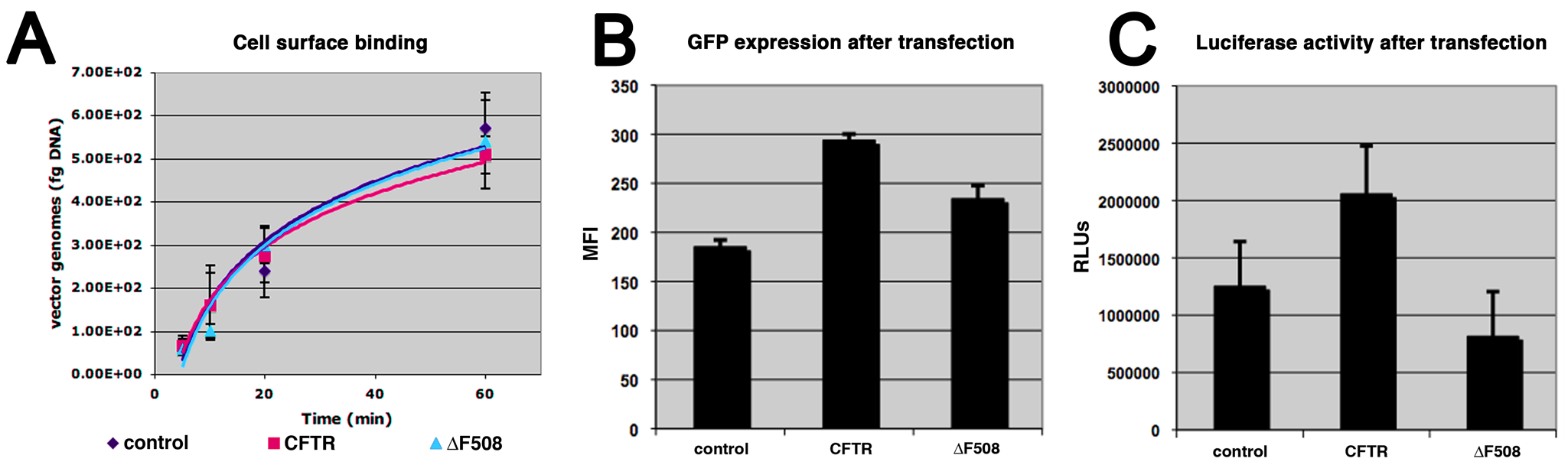

Supplement: Figure S1 — Exploitation during infection likely occurs between virus binding and gene expression. A. Cell surface binding of AAV2. Equal numbers of control wt BHK-21 cells (purple diamonds), cells expressing CFTR (red squares), or ΔF508-CFTR (blue triangles) seeded in 24 well plates were cooled to 4°C to inhibit endocytosis. AAV2 was applied (10,000 vg/cell) for the indicated times, and the DNA was harvested for qPCR quantification of vector genomes. Error bars represent standard deviations from three independent samples. B. Analysis of expression levels after transfection of reporter plasmids by flow cytometry indicating mean fluorescence intensity (MFI) of GFP-positive cells 24 hr after transfection of TR-eGFP in control cells, cells expressing CFTR, or ΔF508-CFTR C. Luciferase assay of reporter expression in control cells, CFTR-expressing cells, or ΔF508-CFTR expressing cells, 24 hr after transfection with a TR-Luc plasmid. Error bars represent standard deviations from three samples. (TIF) [file ppat.1002053.s001.tif]

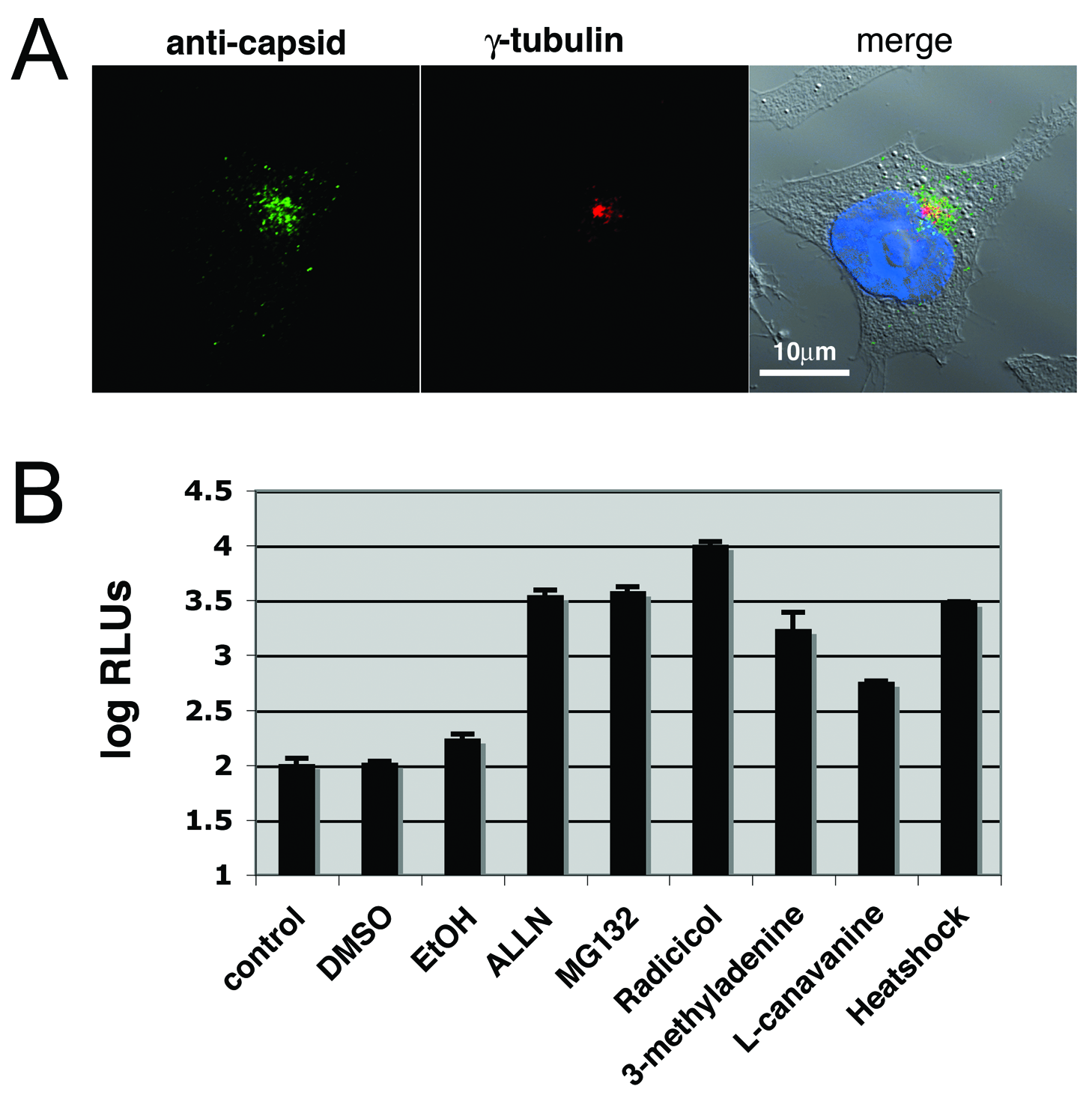

Supplement: Figure S2 — Subcellular stress pathways in trafficking and transduction. A. Confocal immunofluorescence microscopy depicts AAV2 capsids (i, green) trafficking near the microtubule organizing center (ii - γ-tubulin, red) at 4 hr post-infection in HeLa cells. B. Luciferase assay of transduction showing several treatments that influence misfolded protein processing positively impact AAV2 transduction. AAV2 capsids (10,000 vg/cell) were administered concurrently with either vehicle controls (DMSO or EtOH), or ALLN (40 µM), MG132 (2 µM), Radicicol (10 µM), 3-methyladenine (10 mM), or administered directly after treating with L-canavanine (10 mM, 12 hr), or during heat shock (4 hr at 42.5°C). DMSO and EtOH did not significantly influence expression. The proteasome inhibitors ALLN and MG132 increased transduction between 30 and 40-fold. The Hsp90 inhibitor, radicicol, known to perturb chaperone activity and induce a heatshock-like response [79], increased transduction by roughly 100-fold. 3-methyladenine, an inhibitor of autophagy [80], [81], augmented transduction by at least 20-fold. L-canavanine, an amino acid analogue of arginine, induces widespread protein misfolding [82] and increased AAV transduction 5- to 10-fold. Heat shock also increased transduction as previously documented [51]. Luciferase activity was measured 24 hr post-infection. (TIF) [file ppat.1002053.s002.tif]

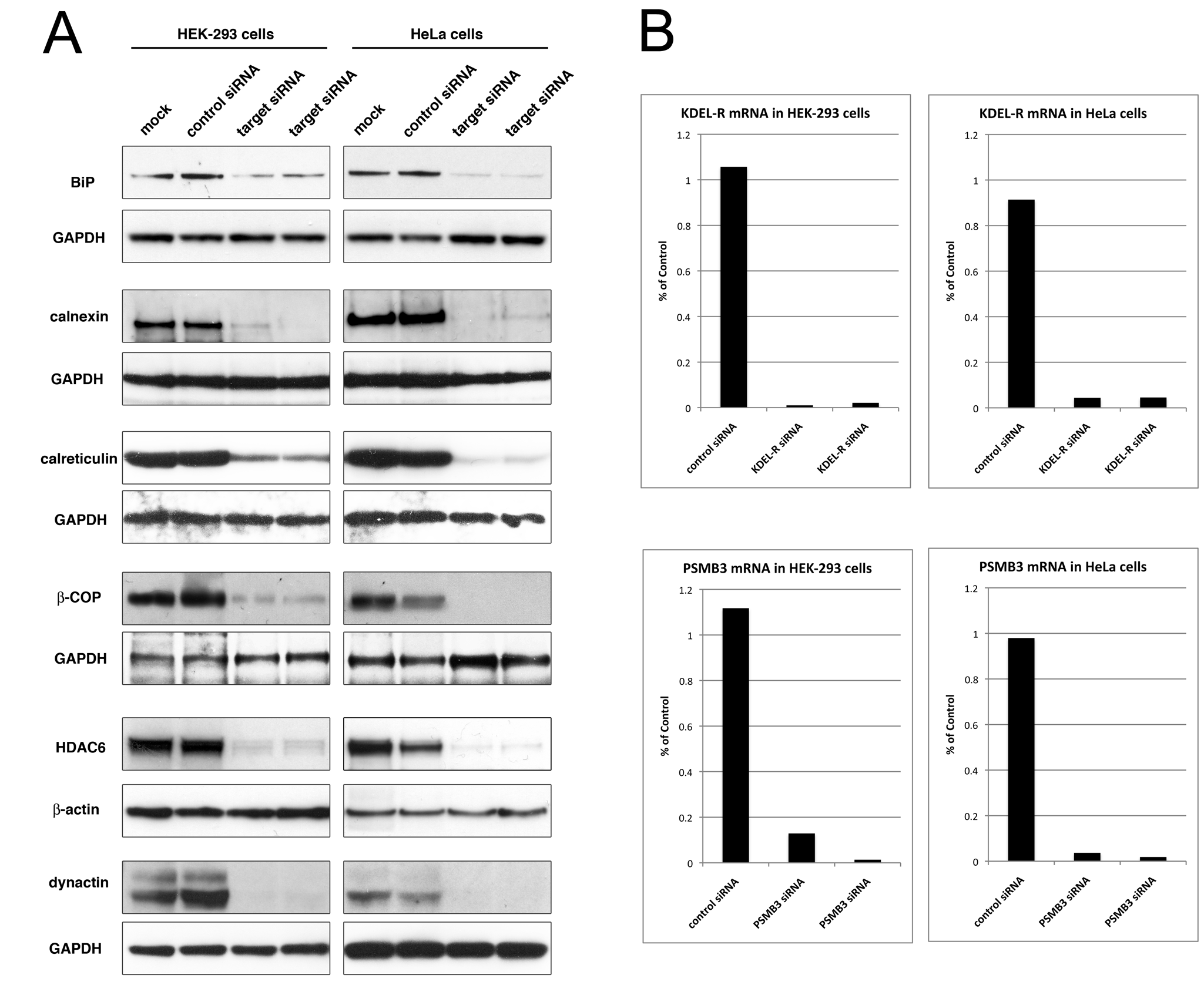

Supplement: Figure S3 — siRNA-mediated knockdown of secretory pathway components or proteins involved in CFTR processing. A. Representative Western blots demonstrating levels of target protein reduction compared to GAPDH or β-actin controls after siRNA transfection (∼48 hr), corresponding to time of infection. B. For those targets where a reliable antibody was not available for Western blot (KDEL-R and PSMB3), we quantified levels of mRNA by RT-qPCR, normalized to mRNA for GAPDH. Transfections for mock conditions (transfection reagent alone), control (non-targeted siRNA), or two target siRNAs were performed in HEK-293 and HeLa cells according to procedures described in Materials and Methods. (TIF) [file ppat.1002053.s003.tif]

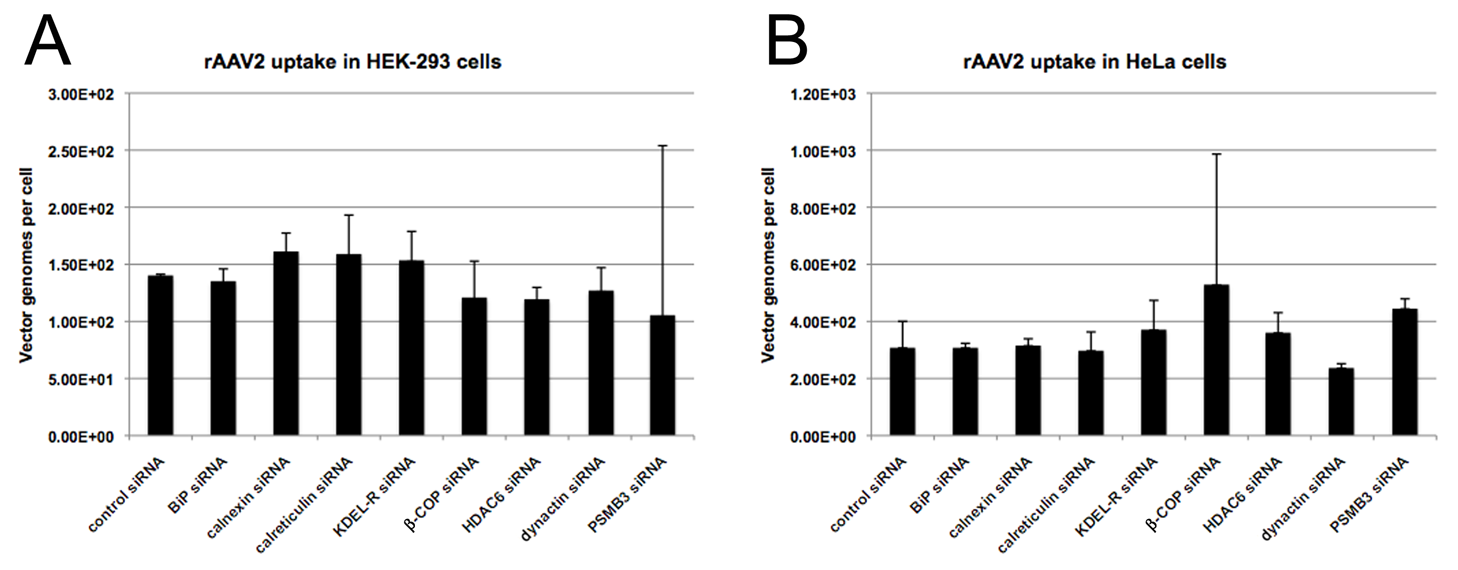

Supplement: Figure S4 — Chymotrypsin-like proteasome activity in cells transfected with siRNA. Two days following transfection with non-targeted control or indicated targeted siRNAs, HEK-293 (A) or HeLa cells (B) were split to 20,000 cells/well in 96 well plates and tested for proteasome activity using a cell-based luciferase assay (Promega G8660) according to the manufacturer's instructions. Data shown are representative of two experiments with standard deviations from three independent samples. (TIF) [file ppat.1002053.s004.tif]

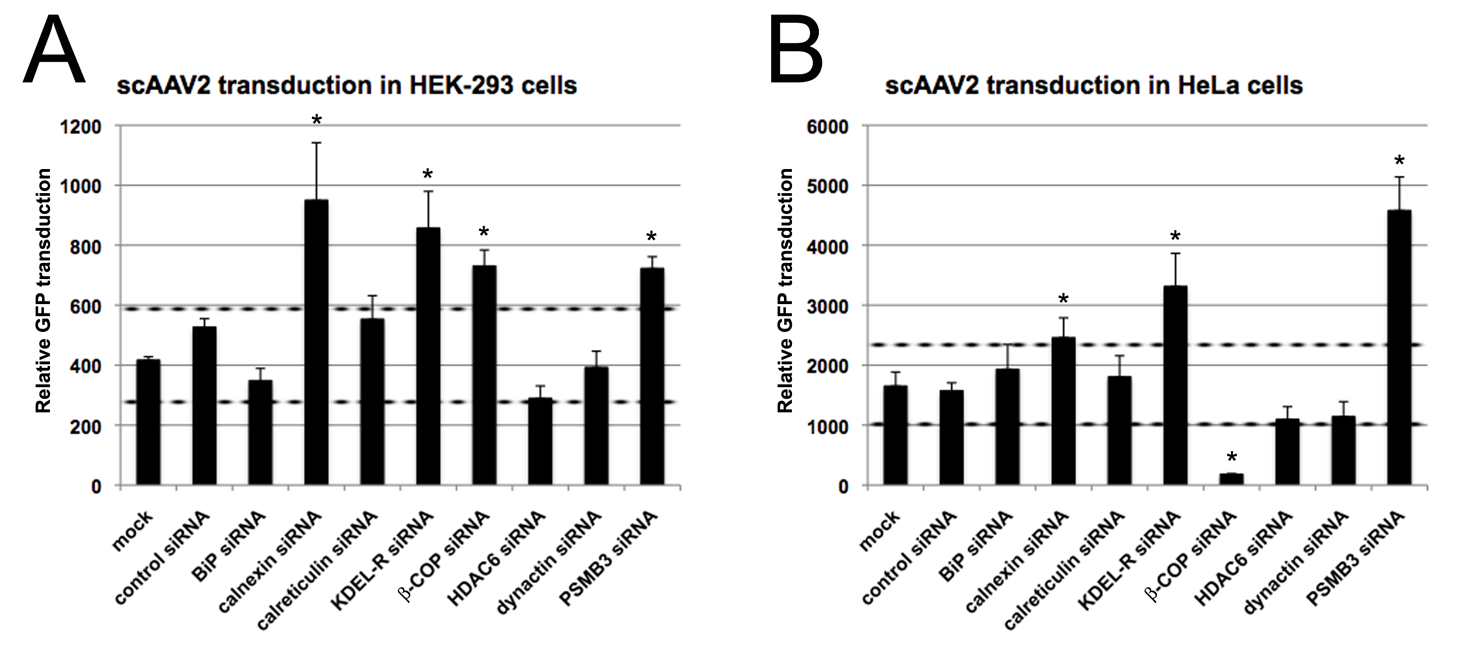

Supplement: Figure S5 — Quantification of viral uptake in cells transfected with siRNA. Between 36 and 48 hr following transfection with non-targeted control or indicated targeted siRNAs, HEK-293 (A) or HeLa cells (B) were inoculated with rAAV2 (5,000 vg/cell or 10,000 vg/cell, respectively) for 24 hr. Total DNA was harvested from cells by DNeasy preparation according to procedures in Materials and Methods and viral genomes were quantified by qPCR which were normalized to GAPDH levels. Data shown represent averages with standard deviations from two independent experiments. (TIF) [file ppat.1002053.s005.tif]

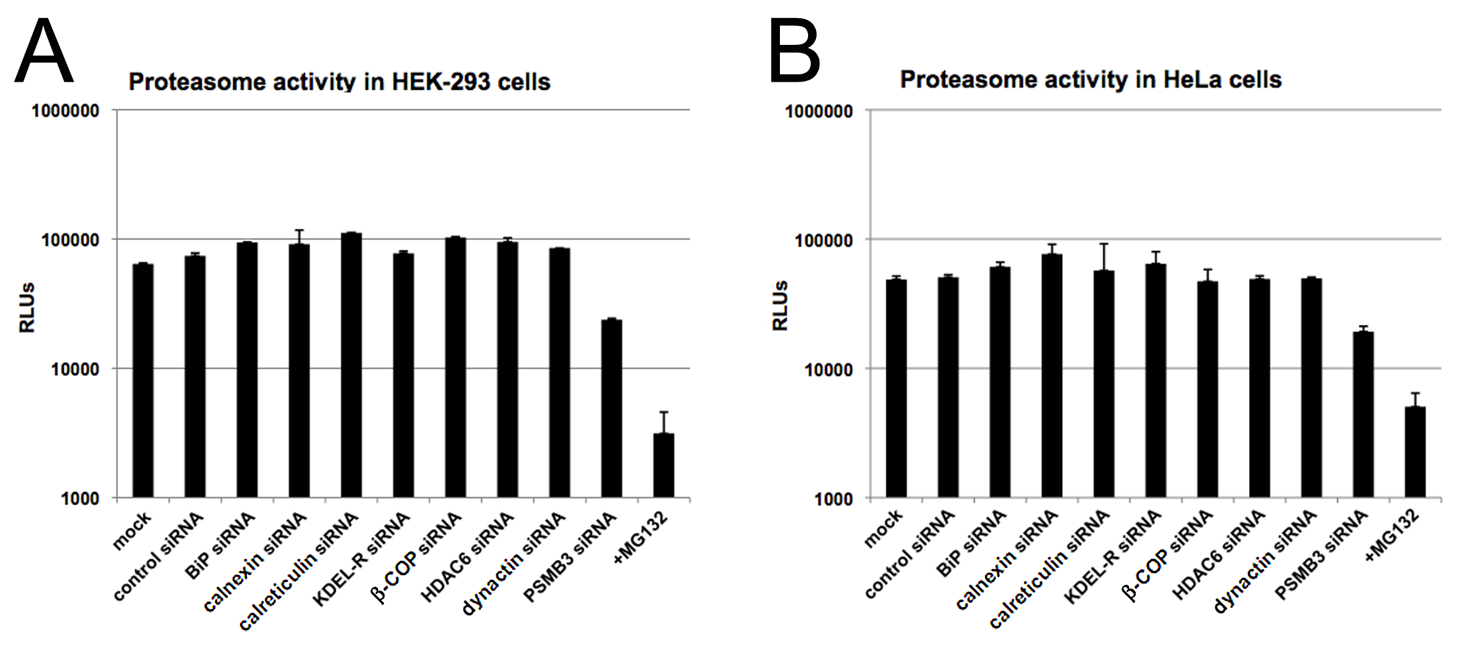

Supplement: Figure S6 — Transduction efficiency of self-complementary rAAV2 in cells transfected with siRNA. Transduction efficiency of self-complementary AAV2 (scAAV2) following siRNA-mediated knockdown of proteins in HEK-293 cells (A) or HeLa cells (B). A margin of error for nonsignificant perturbations of infection (roughly ±30% relative to controls) is indicated by dashed lines. Cells were mock transfected or transfected with non-targeting control siRNA, or siRNA targeting BiP, calnexin, calreticulin, KDEL-R, β-COP, HDAC6, dynactin, or PSMB3. Between 36 and 48 hr after transfection, cells were infected with scAAV2-GFP (50 vg/cell, HEK-293; 100 vg/cell, HeLa) and fixed for flow cytometry 24 hr post-infection. Data is reported as the percentage of cells infected times mean fluorescence intensity. Data is representative of two independent experiments with error bars indicating standard deviation. Samples that were statistically different from controls are marked (*p<0.05). (TIF) [file ppat.1002053.s006.tif]

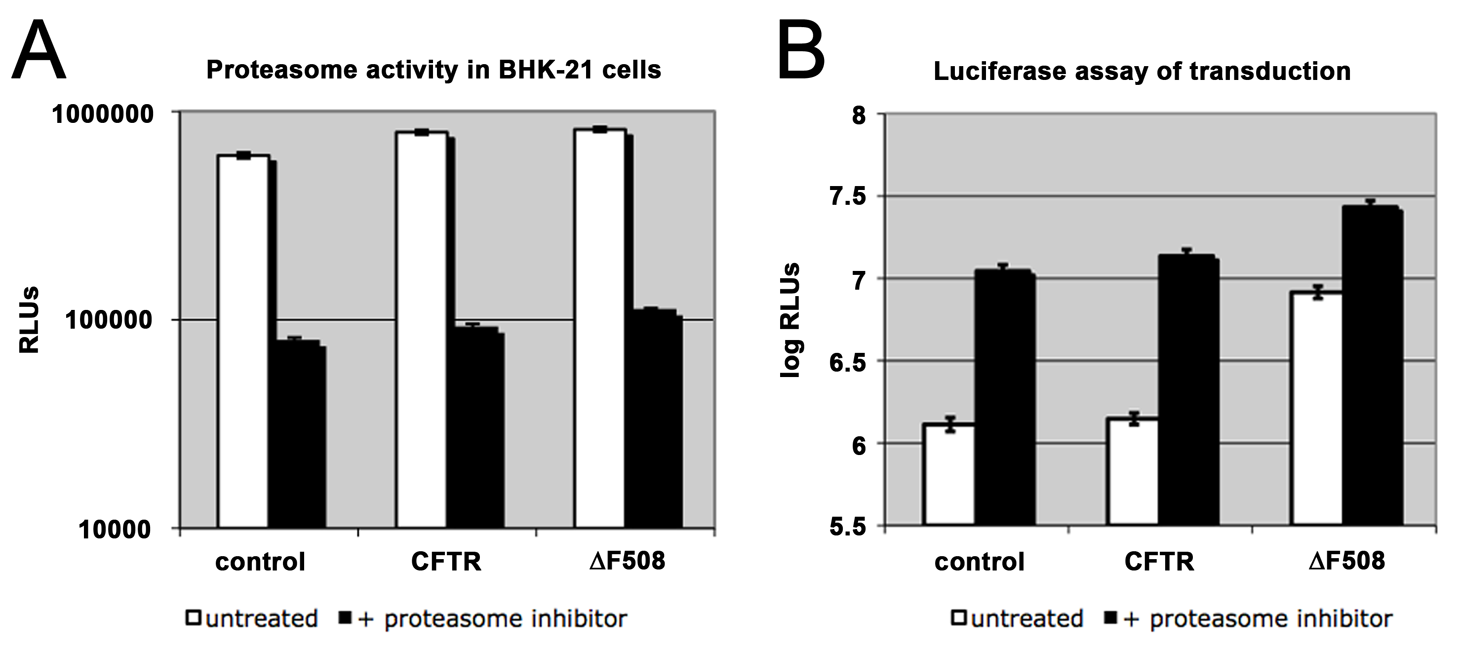

Supplement: Figure S7 — Influence of the ubiquitin/proteasome system on AAV infection in models of CF. A. Luciferase assay of proteasome activity in BHK-21 cells with or without proteasome inhibitor (MG132, 2 µM). B. Luciferase assay of AAV2 transduction in HEK-293 cells with or without proteasome inhibitor. AAV2 capsids (10,000 vg/cell) were administered to equal numbers of control, CFTR, or ΔF508 cells in 24 well plates and scored for transduction 24 hr after inoculation. Proteasome inhibition potentiates infection efficiency in each cell type, but is less effective in ΔF508 cells. (TIF) [file ppat.1002053.s007.tif]

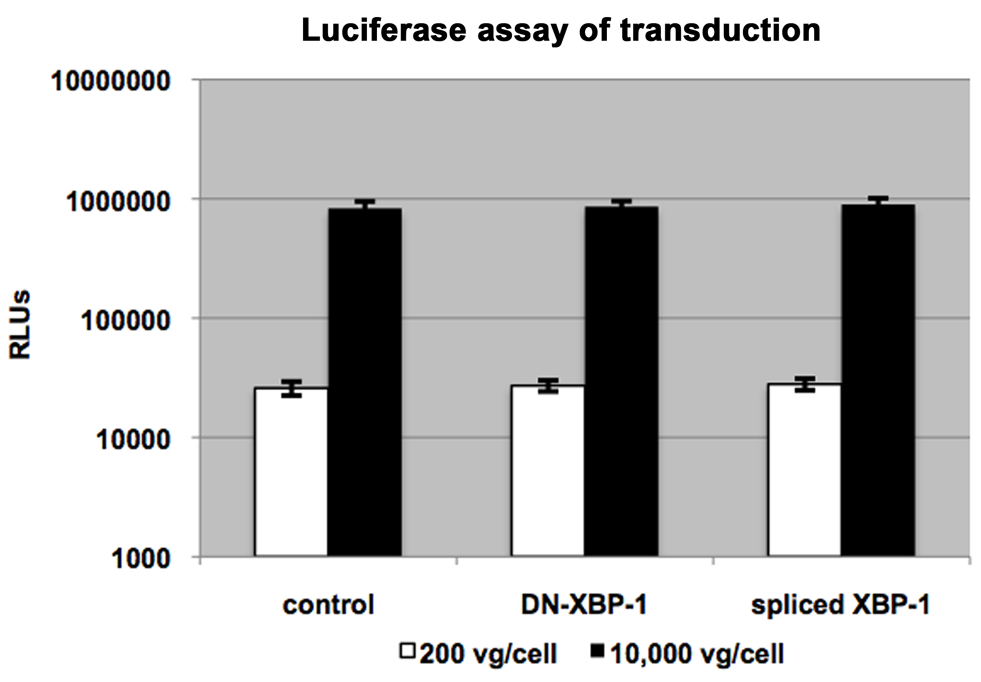

Supplement: Figure S8 — Influence of XBP-1 splicing and the unfolded protein response on rAAV2 transduction. Transformed human bronchial epithelial cells (16HBE14o-, control), or 16HBE14o- cells stably expressing dominant negative XBP-1 (DN-XBP-1), or expressing constitutively active XBP-1 [spliced XBP-1; (Lee, A. H., Iwakoshi, N. N., and Glimcher, L. H. (2003.) Mol. Cell. Biol. 23 7448–7459)], were inoculated with rAAV2 at the indicated particle numbers. After 24 hr a luciferase assay of transduction was performed to measure efficiency of AAV2-mediated gene delivery. Error bars represent standard deviations from four independent samples. (TIF) [file ppat.1002053.s008.tif]
